# A paracrine network regulates the cross-talk between human lung stem cells and the stroma

**DOI:** 10.1038/ncomms4175

**Published:** 2014-01-16

**Authors:** E. Josue Ruiz, Feride Oeztuerk-Winder, Juan-Jose Ventura

**Affiliations:** 1 SCI (Wellcome Trust-Medical Research Council Stem Cell Institute, University of Cambridge), Tennis Court Road, Cambridge, CB2 1QR, UK

## Abstract

The signals that regulate stem cell self-renewal and differentiation in the lung remain elusive. Lung stem cells undergo self-renewal or lineage commitment to replenish tissue, depending on cross-talk with their environment. This environment, also known as the niche, includes mesenchymal and endothelial tissues. Here we define molecular mechanisms involved in the interaction between human lung Lgr6+ stem cells (LSCs) and fibroblasts in a functional microenvironment. We reveal a central role for p38α MAPK in establishing and maintaining such cross-talk, acting in both cell types. In LSCs, p38α induces the expression of SDF-1, which activates the stroma. p38α is essential for fibroblast activation and induction of cytokine expression, in particular TNFα. This paracrine network induces a hierarchical activation leading to the recruitment of endothelium, establishing a functional microenvironment. Disruption of this cross-talk abrogates proper LSC differentiation *in vivo* and may lead to lung dysfunction and disease.

## Abstract (web summary)

The human lung contains Lgr6-positive progenitor cells, which have the potential to generate multiple epithelial lineages in cell explant studies. Here, the authors present a role of p38a MAPK signalling in regulating the cross-talk between this lung progenitor cell population and the stromal and endothelial cells comprising their niche microenvironment.

## Graphical abstract

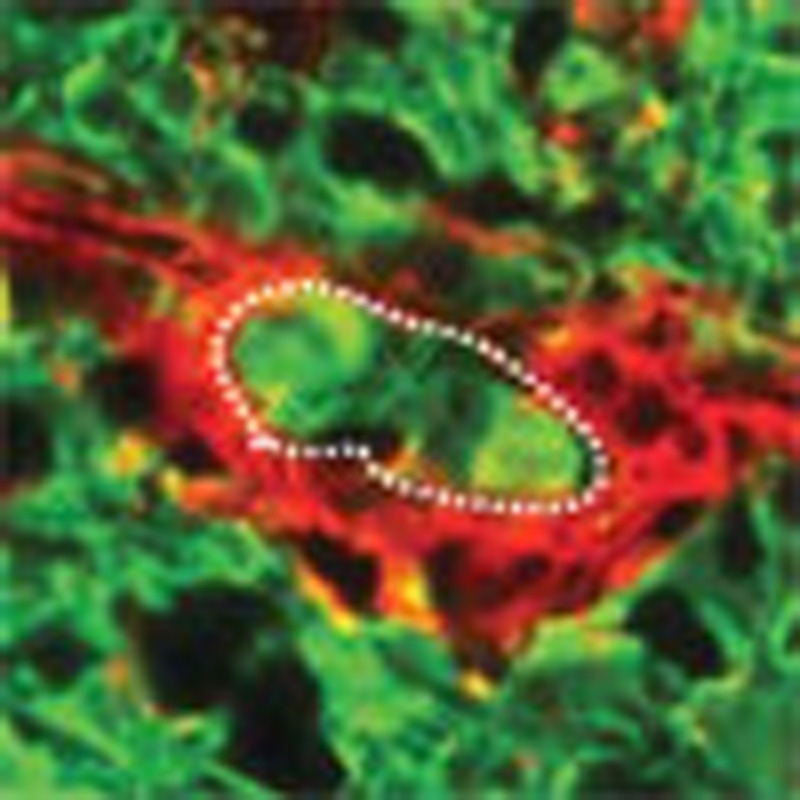

The stromal microenvironment plays a fundamental role in the regulation of tissue homeostasis and the promotion of pathological processes. The stem cell ‘niche’ allows the maintenance of multipotency and regulates cell division^1^. The niche is a microenvironment that includes the extracellular matrix, cell contacts and a broad number of autocrine and paracrine signals and hormones^2^,^3^. Altogether, these contributors regulate the stem cells to either remain in the niche, divide symmetrically or asymmetrically, or migrate from the niche and differentiate into either transient progenitors or terminally differentiated cells^4^.

In the lung, several niches have been described that harbour different adult multipotent stem cells involved in the turnover of distinct anatomical areas of the lung. There are different types of stem cells in the trachea (submucosal gland stem cell), bronchi (basal cell) and bronchioles (neuroendocrine bodies)^5^. Recently, a group of cells has been reported as putative progenitors for the mouse bronchioalveolar area, with the potential to differentiate into Clara or Alveolar (type 1 or 2) cells^6^.

We previously isolated a population of mouse bronchioalveolar cells based on negative selection for non-epithelial markers and sorting for Sca-1/E-Cad-positive cells^7^. Furthermore, we have recently characterized a clonally derived population of human lung Lgr6+ stem cells (LSCs) from the distal lung, which express E-Cadherin and Lgr6, but not endothelial, mesenchymal or hematopoietic markers (CD34^−^/CD73^−^/CD45^−^/PECAM^−^). The stem cell potential of these cells has been confirmed using different *in vitro* and *in vivo* assays, such as kidney capsule engraftments and culture of lung explants^8^. In kidney grafts, LSCs are able to recapitulate a bronchioalveolar epithelium and also to recruit connective (Vimentin^+^) and endothelial (CD73^+^) cells to create a functional environment^8^.

The role of paracrine signals in the maintenance of stem cell niches is well known. Activation of stromal cells, and specially fibroblasts, to induce their migration and production of other paracrine signals plays an essential role in niche formation in cancer and homeostasis^9^,^10^. Simultaneously, molecular signals released by the stroma control cell division and fate determination in stem cells^11^. Stromal regulation is pivotal for proper lung homeostasis and the existence of a niche is necessary to create a functional adult tissue with a turnover potential^12^.

The balance in cross-talk between signals from the stem cells and signals from the stroma may also be a determinant for the proper regeneration of the bronchioalveolar epithelium after injury^13^. Failure to maintain the right balance may lead to pathological processes (for example, lung fibrosis, cancer metastasis), in which inflammatory signalling promotes expansion of the stromal compartment while preventing epithelial differentiation and functional tissue repair^14^.

Here, we delineate how the molecular interactions of a paracrine signalling circuit of cytokines and chemokines, released by LSCs and stromal fibroblasts, create a self-maintained functional microenvironment both *in vitro* and *in vivo*. At the centre of this circuit, acting as intracellular mediators of paracrine cues in both LSCs and fibroblasts, is the p38α MAPK signalling pathway. This kinase pathway co-ordinates the release and response to cytokines in both cell types, and it is necessary for LSCs to establish and maintain a bronchioalveolar environment *in vivo*.

## Results

### LSCs interact with their environment

**Figure 1:**
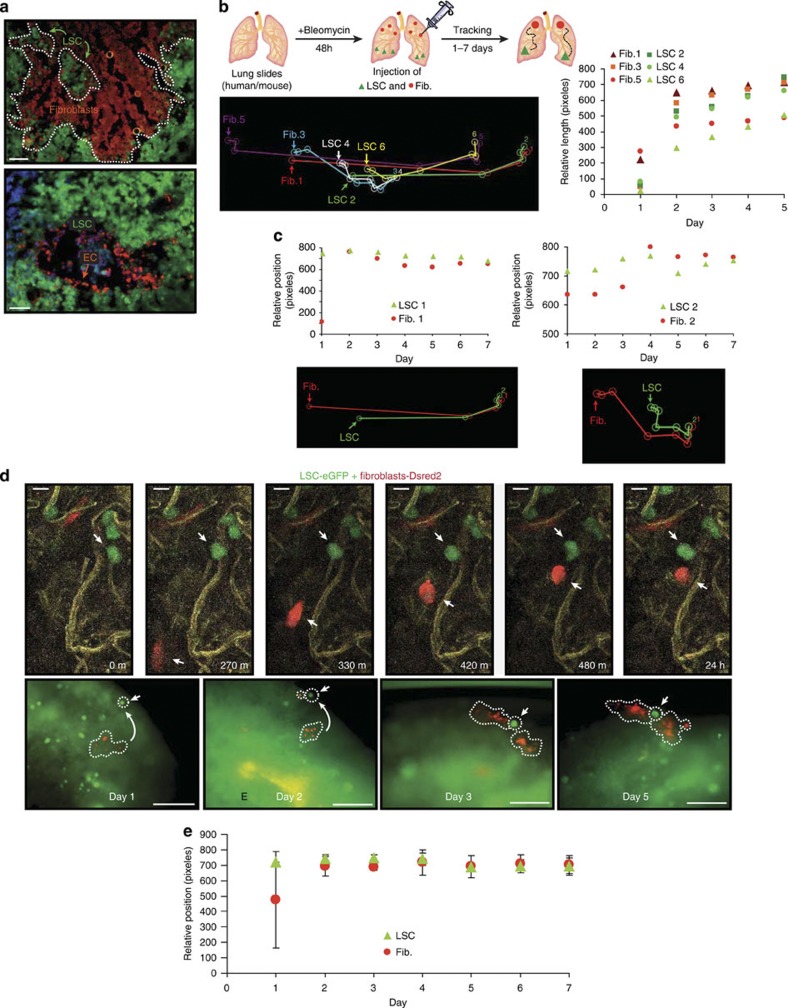
Analysis of stromal cell recruitment by lung stem cells. (**a**) LSCs (GFP-labelled) engraft in the kidney capsule and recruit fibroblasts (Vimentin^+^) and endothelial cells (CD73^+^) (red). Note that fibroblasts surrounded the LSCs. Scale bars: 100 μm. (**b**) General strategy to track the movement of the cells. Schematic tracing of three pairs of LSC-fibroblasts injected in *ex vivo* lung tissue from different distances at starting point (arrows). Relative length graph showing the spatial location of the LSC-fibroblasts injected during a 5-day period. (**c**) Graphs showing the relative positioning and schematic tracing of single pairs of LSC-fibroblast cells from different distances at starting point (arrows). (**d**) Real-time tracing of LSCs (green) and fibroblasts (red) over a 24 h (upper panel, scale bars: 20 μm) or 5 days period (lower panel, scale bars: 200 μm). (**e**) Average of the relative positioning between injected fibroblasts and LSCs. *N*=35, from 4–5 experiments±s.e. of the mean s.e.m.

We have described a clonally derived enriched human Lgr6+ population with the potential to differentiate *in vitro* and *in vivo* into all bronchioalveolar mature cell types, named as Lgr6+ stem cells (LSCs from here on). These cells are able to produce a bronchioalveolar epithelium in an alien environment when injected under the kidney capsule of nude mice^8^. This epithelium contains connective and endothelial tissue (Fig. 1a). Interestingly, we observed that a discrete population of LSCs is surrounded by fibroblasts (Vimentin^+^) mimicking a niche, suggesting that LSCs are able to recruit stromal cells to create their own microenvironment (Fig. 1a).

### Reciprocal modulation between LSCs and fibroblasts

To functionally test the ability of LSCs to recruit stromal cells, we used lung slices treated with bleomycin, to remove all kind of cellular constituents, including bronchiolar, alveolar, mesenchymal and endothelial cell types (Supplementary Fig. 1 and 8). *Ex vivo* bleomycin has been used for many years in lung explants as a model of lung toxicity *in vitro*^15^,^16^. As a DNA-damaging and ROS-induced apoptotic agent^17^, it kills most cell types, without any inflammatory response, producing a physiological scaffold that allows studying the process followed by LSCs to promote the recruitment of stromal cells. We injected differentially labelled LSCs and fibroblasts at different sites to test the range of distance-dependent paracrine cross-talk. The distance was determined by monitoring the locations and relative positions of individual LSC and fibroblast cells over a period of 7 days, allowing us to quantify the recruitment of fibroblasts to the proximity of LSCs (Fig. 1b–e and Supplementary Movie 1). Cell tracking was performed for each cellular type within the region of analysis with an ImageJ software complemented with a MtrackJ plugin. We observed a maximum distance of 10^3^ μm for the paracrine signals to engage LSC recruitment of fibroblasts (Supplementary Fig. 2A), in either bleomycin-treated or non-treated lung explants (Supplementary Fig. 2B). The dynamics of the recruitment varies, and it may depend on paracrine factor gradients around different LSCs (Fig. 1d,e). Proliferation of LSCs and fibroblasts is promoted only after they get in close proximity (Fig. 1d, and Supplementary Fig. 2A). Fibroblasts migrate to LSCs vicinity but do not engage in direct contact at early time points (Fig. 1d). However, at later time points LSCs are surrounded by fibroblasts (Fig. 1d and Supplementary Fig. 2B), recapitulating the kidney graft results (see Fig. 1a).

Thus, these findings suggest that there is a paracrine cross-talk between LSCs and fibroblasts inducing the recruitment of the latter, and recruited fibroblasts enclosing LSCs could function as putative niche cells.

**Figure 2:**
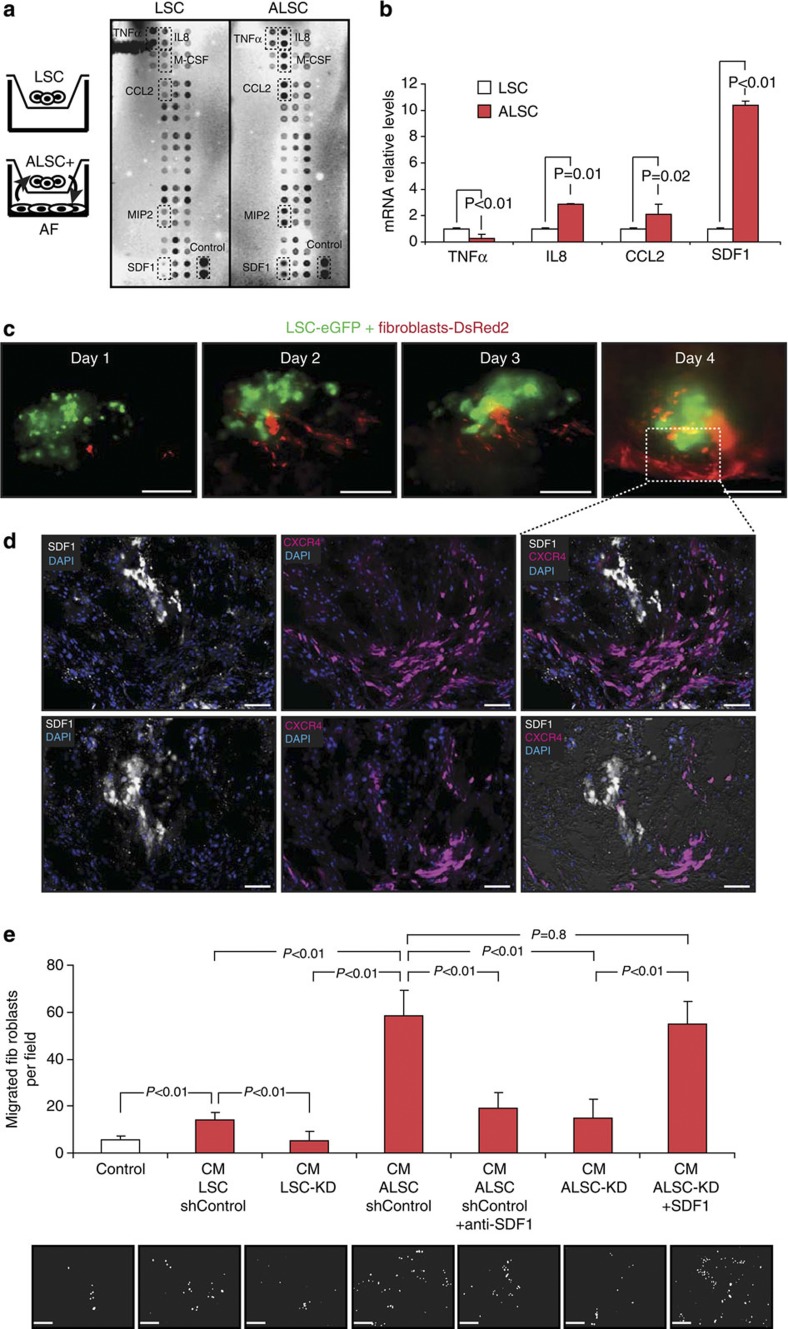
SDF-1 released by activated LSCs is the key paracrine factor to recruit stromal fibroblasts. (**a**) Protein array showing differential cytokine release by activated LSCs (ALSC) when co-cultured with fibroblasts. (**b**) The mRNA relative levels of different cytokines confirm their upregulation in ALSCs. (**c**) Time-lapse tracing of fibroblast (red) recruitment by LSCs (green) in lung explants. Scale bars, 200 μm. (**d**) Serial sections images captured at day 4 showing expression of SDF-1 (white) and CXCR4 (purple) by LSCs and fibroblasts, respectively. Scale bars, 50 μm. (**e**) Fibroblast migration induced by conditioned medium (CM) from ALSCs (expressing control shRNA) (line 4) is prevented by addition of an anti-SDF-1 antibody (line 5) or by using CM from LSCs lacking SDF-1 (ALSC-KD) (line 6). Addition of SDF-1 recombinant protein into CM from ALSC-KD cells rescued their potential to recruit fibroblasts (line 7). Scale bar, 200 μm. All results (**b** and **e**) are the mean±s.e.m. of 4–5 triplicate experiments. *P*<0.01 values were defined as statistically significant, as analysed by one-way ANOVA.

To begin defining the mechanisms involved in the paracrine signalling induced as part of the interplay between LSCs and lung fibroblasts, we used Boyden chambers. A comparative analysis of the factors released into the medium using a cytokine array showed a number of changes in the paracrine signal profile released by LSCs and fibroblasts when cultured either alone or in close proximity to each other (Fig. 2a, Supplementary Fig. S2C and Supplementary Table 1 and 2). LSCs co-cultured with fibroblasts were considered as activated-LSCs (ALSCs). Cytokines found to be released differentially by ALSCs were involved in the inflammatory response (TNFα), vascularization (IL8, MIP2) or chemoattraction (SDF-1) (Fig. 2a and Supplementary Fig. 2C). These variations in cytokine release were transcriptionally regulated, as cytokine mRNA expression correlated with protein levels (Fig. 2b). Especially interesting was the increase in SDF-1 levels induced in ALSCs. This chemokine is essential in maintaining the hematopoietic stem cell niche, although usually from mesenchymal or immune cell origin^18^. Furthermore, in the co-cultures, activated fibroblasts (AFs) showed an increased in the expression of both SDF-1 receptors, CXCR4 and CXCR7 (Supplementary Fig. 2D).

### SDF-1 is the key paracrine factor to recruit stromal cells

To begin defining whether SDF-1 is required for fibroblast recruitment, LSCs and fibroblasts were injected into human lung explants, and then we analysed the expression of SDF-1 in LSCs and CXCR4 expression in fibroblasts. Serial sections of the explants were captured by immunofluorescent microscopy for the unambiguous identification of both proteins. Importantly and consistent with the above results, when fibroblasts are recruited by LSCs (Fig. 2c), the stem cells expressed SDF-1 and the fibroblasts expressed the CXCR4 receptor (Fig. 2d).

Using transwell chambers, we also observed that conditioned medium (CM) from ALSCs induced the migration of fibroblasts more strongly than CM from LSCs cultured alone (Fig. 2e, compare lines 2 and 4). Migration was specifically activated, independently of proliferation, which was not affected. To test whether SDF-1 released by ALSCs is responsible to induce fibroblast migration, CM from ALSCs was incubated with a neutralizing antibody for SDF-1. We found that blocking SDF-1 activity prevented the ability of the ALSCs to induce fibroblast migration (Fig. 2e, see lines 4 and 5). To confirm this observation, LSCs expressing short hairpins for SDF-1 (LSC-KD) were generated (Supplementary Fig. 2E) and the CM from either LSC-KD cultured alone or co-cultured with fibroblasts (ALSC-KD) was used as a chemoattractant. As expected, knocking down SDF-1 in ALSCs (Supplementary Fig. 2F) also inhibited fibroblast migration (Fig. 2e, see lines 4 and 6). Finally, confirmation of the chemoattractant role of SDF-1 was shown by rescuing recruitment of fibroblasts by the addition of recombinant SDF-1 protein to the CM from ALSC-KD (Fig. 2e, see lines 4, 6 and 7).

**Figure 3:**
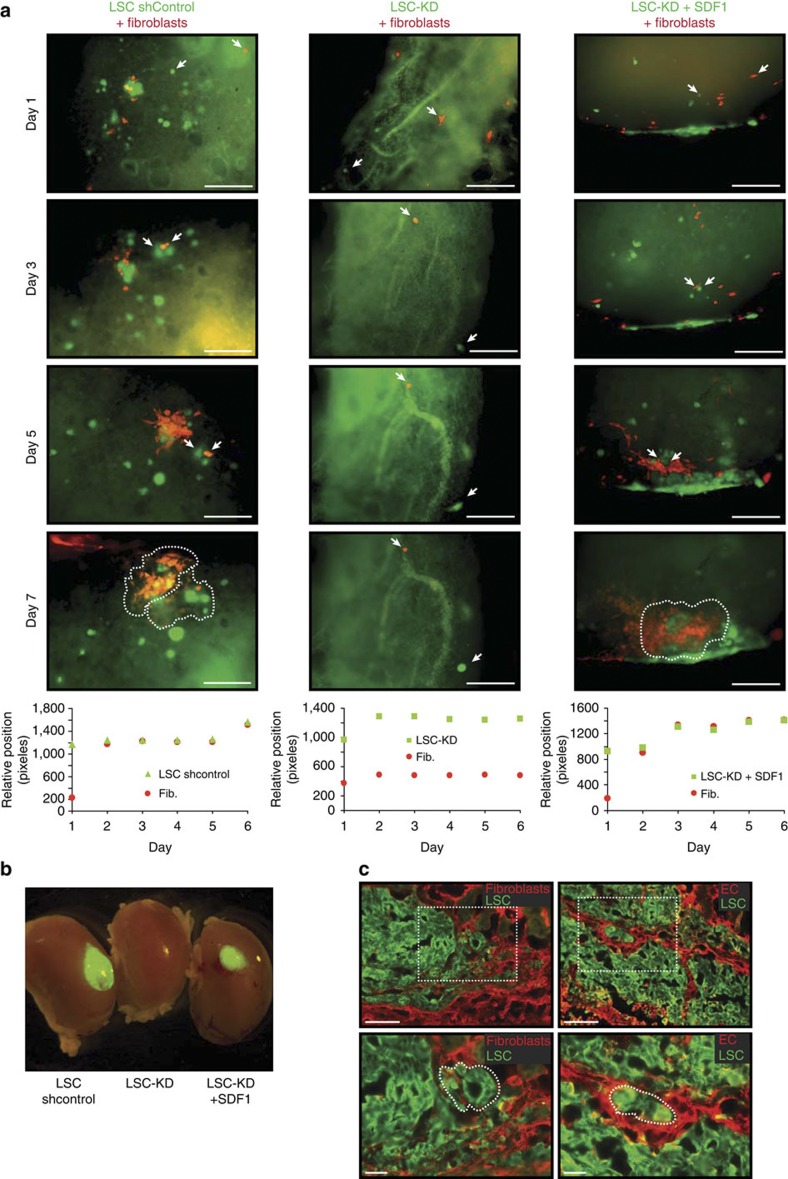
Tracking of SDF-1 recruitment of stromal cells *in vivo*. (**a**) Time-lapse image cell tracking of fibroblasts (red) and LSCs (expressing control shRNA), LSC-KD or LSC-KD+SDF-1 cells (recombinant SDF-1, embedded in the matrigel, was used to inject the cells) injected into lung explants. Arrows indicate the cells shown in the lower graphs, which represent their relative position. Scale bars, 200 μm. (**b**) LSCs shControl and LSC-KD+SDF-1, but not LSC-KD cells engraft under the kidney capsule at 2 weeks. LSC-KD cells overexpressed a SDF-1 mRNA resistant to shSDF-1 knockdown. (**c**) Immnuofluorescence of kidney engraftments of injected LSC-KD+SDF-1 cells showing the recruitment of fibroblasts (Vimentin^+^) and endothelial cells (CD73^+^). Note that either fibroblasts or endothelial cells surround a discrete population of LSCs. Upper panel, scale bar, 200 μm. Lower panel, scale bar, 100 μm.

On the basis of this evidence, we hypothesized that the inhibition of SDF-1 would result in the impairment of fibroblast migration in lung explants. To test this hypothesis, we injected fibroblasts together with either LSCs expressing a short hairpin control or LSC-KD into human lung explants. In agreement with previous results, LSCs expressing a shControl were able to recruit fibroblasts (Fig. 3a). However, the injection of LSCs lacking SDF-1 expression confirmed the absence of the necessary paracrine cross-talk to recruit fibroblasts via SDF-1 (Fig. 3a, middle panel, and Supplementary Fig. 3A, B). Fibroblasts recruitment could be rescued by overexpression of a SDF-1 mRNA resistant to shSDF-1 knockdown or embedding SDF-1 protein in the matrigel carrying the LSC-KD cells (Fig. 3a, right panel, and Supplementary Fig. 3C,D). Furthermore and consistent with the above results, recruited fibroblasts are able to enclose LSCs.

Finally, kidney capsule engraftments were used to test, *in vivo*, the observed *in vitro* role of SDF-1 in the recruitment of stromal fibroblasts. We found that LSCs lacking SDF-1 (LSC-KD) failed to produce grafts under the kidney capsule (Fig. 3b and Supplementary Fig. 3E). However, again it could be rescued by overexpression of shSDF-1 resistant mRNA (Fig. 3b and Supplementary Table 3) or embedding SDF-1 recombinant protein in the matrigel carrying the LSC-KD cells (Supplementary Fig. 3E,F). The inability of the LSC-KD cells to engraft was independent of their proliferative potential (Supplementary Fig. 3G). Interestingly, the engraftments showed that LSC-KD cells, rescued by shSDF-1 resistant mRNA, were able to recruit stromal cells from the host tissue (Fig. 3c). Furthermore, we also found that a small population of LSCs was surrounded by fibroblasts (Vimentin^+^) or endothelial cells (CD73^+^) recapitulating a niche (Fig. 3c). The mouse origin of the stroma in the graft was confirmed as it did not express GFP and was negative for an anti-human mitochondrial antibody (Supplementary Fig. 3H).

Together, these data suggest that SDF-1 released by LSCs has an essential role in the recruitment of stromal cells.

### Regulation of SDF-1 expression and secretion by TNFα and TGFβ

**Figure 4:**
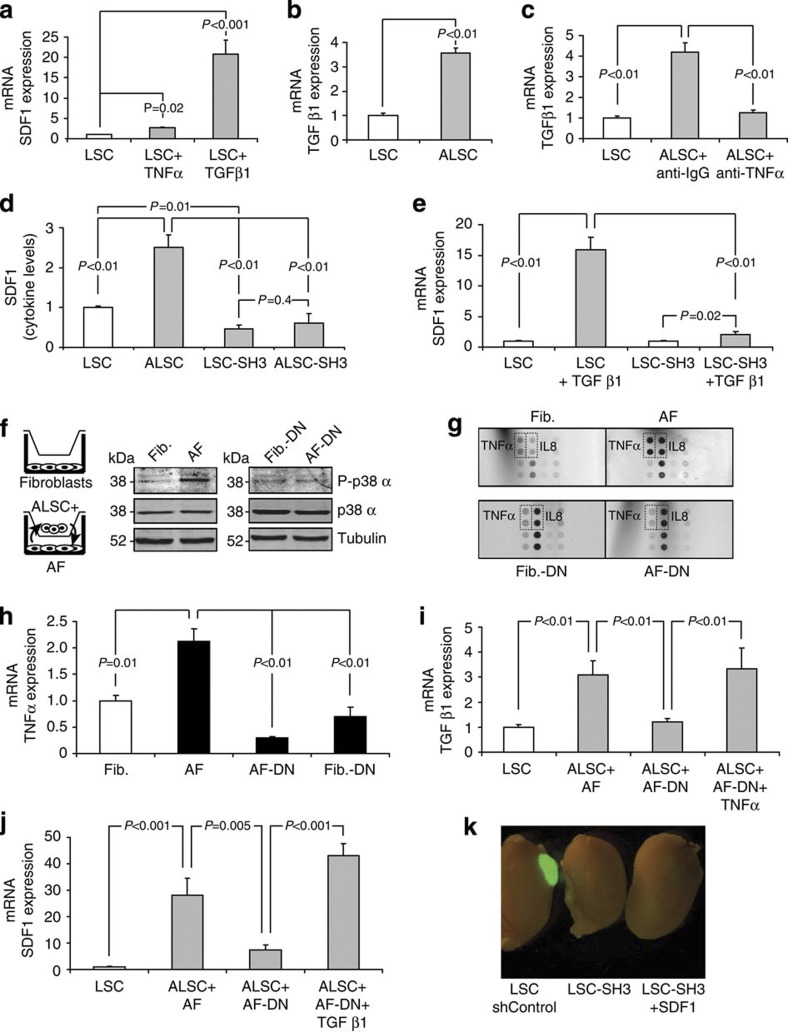
Paracrine cross-talk between LSCs and fibroblasts includes SDF-1 expression in ALSCs and fibroblast activation mediated by p38α. (**a**) TGFβ promotes SDF-1 mRNA expression in LSCs. (**b**) Fibroblasts induced TGFβ expression in ALSCs. (**c**) TGFβ mRNA expression in ALSCs triggers by paracrine TNFα as it can be prevented by adding a blocking antibody. (**d**) SDF-1 expression and release by ALSCs are impaired in both LSC-SH3 and ALSC-SH3, which are p38α deficient. (**e**) TGFβ-induced SDF-1 expression in LSCs is mediated by p38α as it is disrupted in LSC-SH3 cells. (**f**) Western blot showing activation of p38α (P-p38α) in co-cultured WT fibroblasts (AF) but not in fibroblasts expressing a dominant negative form of p38α (AF-DN). (**g**) Protein arrays of cytokines (TNFα, IL8) released to the media by WT or p38^DN^ fibroblasts and their correspondent AFs. (**h**) TNFα expression is induced in AFs but not in activated fibroblasts lacking p38α signal (AF-DN). (**i**) Deficient TGFβ expression in ALSCs co-cultured with fibroblasts lacking p38 activity can be restored by TNFα administration. (**j**) SDF-1 mRNA expression is induced efficiently in ALSCs by AFs but not by AF-DN and can be rescued by addition of recombinant TGFβ. All results (**a**–**e**, **h**–**j**) are the mean±s.e.m. of 4–5 triplicate experiments. *P*<0.01 values were defined as statistically significant, as analysed by one-way ANOVA. (**k**) LSCs lacking p38α (LSC-SH3) and LSC-SH3 cells overexpressing SDF-1 are not able to engraft in the kidney capsule.

Having demonstrated the essential role of SDF-1 to recruit stromal cells, we next sought to understand how SDF-1 expression is regulated by stromal fibroblasts. It has been reported that, depending on cellular context, either TGFβ or TNFα signalling pathways can regulate SDF-1 expression^19^,^20^. Thus, SDF-1 regulation in LSCs by TNFα and TGFβ pathways was tested. Although TNFα was able to induce SDF-1 expression, TGFβ was a much stronger activator in LSCs (Fig. 4a). Interestingly, TGFβ levels were increased in ALSCs (Fig. 4b), but not in AFs (Supplementary Fig. 4A), so TGFβ was excluded as the putative paracrine signal used by the fibroblasts to promote SDF-1 expression in LSCs. Nevertheless, AFs expressed and released more TNFα (Fig. 4g, upper panel, and Supplementary Fig. 4B). This cytokine was considered as a candidate to induce the paracrine activation of LSCs. As TGFβ was not of fibroblastic origin, a possible autocrine regulation of SDF-1 expression in ALSCs was considered. To test this possibility, ALSCs were incubated with a neutralizing antibody for TGFβ. We found that blocking TGFβ activity inhibited the ability of the ALSCs to express SDF-1, confirming the autocrine role of TGFβ (Supplementary Fig. 4C).

TGFβ expression can be induced by TNFα through c-Jun activation^21^. Indeed, LSCs responded to exogenous TNFα treatment by activating the JNK/AP-1 pathway (Supplementary Fig. 4D). In addition, we observed a correlation between TNFα-induced TGFβ and SDF-1 expression in LSCs (Supplementary Fig. 4E). The specificity of this correlation was tested using an inhibitor (SP600125) of the JNK pathway, which abrogated both c-Jun phosphorylation and activation (Supplementary Fig. 4F). These results showed that AP-1 activation was required for TNFα-induced SDF-1 expression (Supplementary Fig. 4G), but not for TGFβ expression, suggesting that other pathway controls TNFα-induced TGFβ expression. However, we confirmed that TGFβ expression in ALSCs was completely dependent on TNFα released by fibroblasts, as it could be prevented using a neutralizing anti-TNFα antibody (Fig. 4c).

These results suggest that activated fibroblasts (AFs) release TNFα, which as a paracrine signal induces TGFβ autocrine loop that enhances SDF-1 production in ALSCs.

### p38α regulates LSCs and fibroblasts activation

As a well-known regulator of cytokine production, the role of p38α in the cross-talk between LSCs and the stromal fibroblasts was investigated using two different approaches. p38α signalling was inhibited in LSCs using a shRNA (SH3) against this kinase^8^. LSC-SH3 cells express very low levels of p38α protein and did not activate the p38α pathway (Supplementary Fig. 4H). Unlike ALSCs, cells lacking p38α (ALSC-SH3) did not respond to fibroblasts stimulation by either releasing SDF-1 protein (Fig. 4d) or expressing SDF-1 mRNA (Supplementary Fig. 4I). In addition, recombinant TNFα failed to induce TGFβ expression in LSC-SH3 cells (Supplementary Fig. 4J), suggesting that in ALSCs p38α is essential to activate the TGFβ autocrine loop induced by paracrine TNFα. Furthermore, the direct role of p38α in SDF-1 expression induced by autocrine TGFβ was confirmed, as exogenous TGFβ was insufficient to promote SDF-1 mRNA expression in LSC-SH3 cells (Fig. 4e). The activation of the TGFβ canonical Smad pathway is not affected in LSC-SH3 cells (Supplementary Fig. 4K).

In fibroblasts, the p38α pathway was inhibited using overexpression of a dominant negative form of p38 (p38^DN^)^22^. p38 activity, but not cJun, was induced in fibroblasts after 6 h of co-culture, and, as expected, it was absent in p38^DN^ fibroblasts (Fib.-DN) (Fig. 4f). Moreover, fibroblasts lacking p38α signalling had reduced levels of both TNFα protein and mRNA compared with WT fibroblasts (Supplementary Fig. 4L). Consequently, when co-cultured with LSCs, p38^DN^ fibroblasts (AF-DN) failed to secrete TNFα (Fig. 4g, h and Supplementary Fig. 4M).

The role of p38α in TNFα production by fibroblasts was found to be essential for the cross-talk with stem cells, as AF-DN cells were unable to induce TGFβ expression in ALSCs, but expression could be restored by supplementing the media with exogenous TNFα (Fig. 4i).

Consequently, disruption of this cross-talk by p38^DN^ overexpression, resulted in the loss of SDF-1 expression mediated by autocrine TGFβ signalling in ALSCs, which was rescued by addition of recombinant TGFβ (Fig. 4j). Finally, TNFα-induced paracrine SDF-1 expression in ALSCs could be inhibited by an anti-TNFα antibody that neutralized the TNFα secreted by AFs (Supplementary Fig. 4N).

Together, these findings show a key role for p38α in mediating TGFβ activation and SDF-1 production in ALSCs, and the expression and release of TNFα by activated fibroblasts.

Finally, we confirmed this essential role *in vivo*, as LSCs deficient in p38α signalling (LSC-SH3) are unable to engraft under the kidney capsule of nude mice. Surprisingly, SDF-1 overexpression could not restore kidney engrafting potential in p38α-deficient cells (Fig. 4k and Supplementary Table 4). This could be explained because of the central role of p38α in LSCs differentiation, and not only in stromal cell recruitment, as we have previously shown^8^.

### TNFα triggers ALSCs-mediated fibroblast recruitment

**Figure 5:**
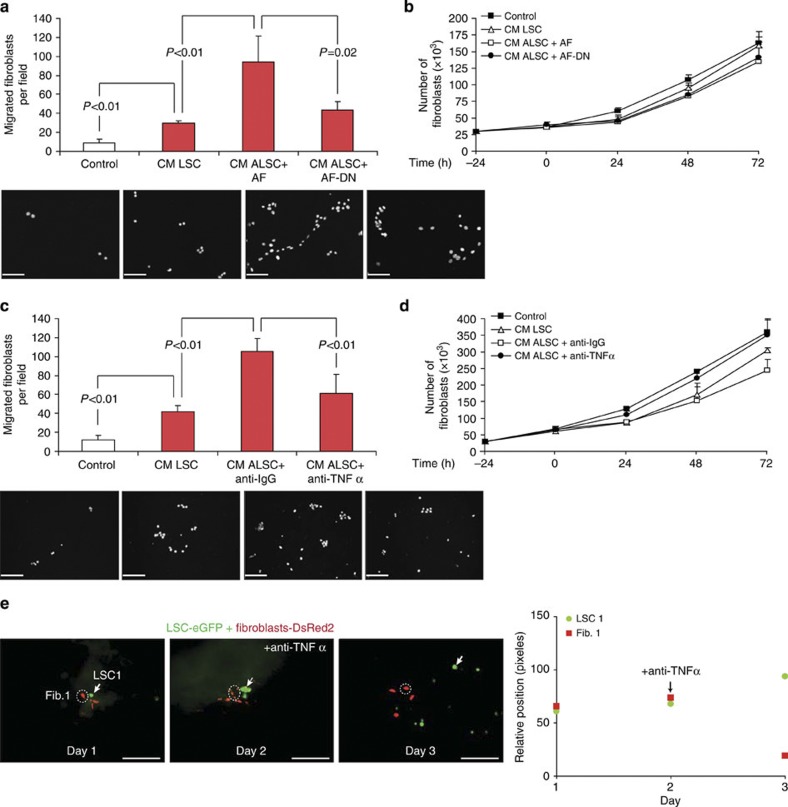
Fibroblast p38α-mediated activation of TNFα expression in cross-talking and ALSCs-dependent induction of fibroblast migration. (**a**) Conditioned medium (CM) from LSCs co-cultured with WT (ALSC), but not with p38^DN^ fibroblasts (ALSC+AF-DN), induces fibroblast migration. Scale bar, 200 μm. (**b**) Fibroblasts proliferate at similar rates when cultured in CM from either LSCs or ALSCs with fibroblasts (WT or p38^DN^). (**c**) Blocking TNFα released by fibroblasts with an anti-TNFα antibody prevents LSCs activation and the ability of the CM to induce fibroblast migration. Scale bar, 200 μm. (**d**) Antibody blocking of fibroblastic TNFα does not affect fibroblast proliferation cultured in LSC-conditioned media. (**e**) Time-lapse images show the engage between fibroblasts (red) and LSCs (green) in *ex vivo* lung explants, which can be disrupted by the addition of an anti-TNFα antibody. Scale bar, 200 μm. All results (**a**–**d**) are the mean±s.e.m. of four triplicate experiments. *P*<0.01 values were defined as statistically significant, as analysed by one-way ANOVA.

As stem cell-induced p38α activation was necessary for fibroblast activation and release of paracrine signals (that is, TNFα), subsequent functional consequences and cellular responses were investigated. In addition to cytokine expression, activation of fibroblasts by ALSCs included induction of migration. As expected, CM from ALSCs induced the migration of fibroblasts more strongly than CM from LSCs either cultured alone or co-cultured with p38^DN^ fibroblasts (Fig. 5a). Migration was independent of proliferation, which was not affected (Fig. 5b). Thus, the migratory response was shown to require previous fibroblastic activation of p38α and TNFα production. In addition, the capacity of CM from ALSCs to promote fibroblast migration was reduced when TNFα from fibroblasts was neutralized with a blocking antibody (Fig. 5c). This was also independent of changes in fibroblast survival (Fig. 5d). Consequently, disruption of the cross-talk between fibroblasts and stem cells by blocking TNFα in lung explants cause the LSCs and fibroblasts to migrate away (Fig. 5e). The effect from TNFα blocking is consistent with the previously shown decrease in SDF-1 levels in ALSCs under this condition (Supplementary Fig. 4M).

Together, these data suggest that activation of LSCs by paracrine TNFα is essential for the subsequent ability of ALSCs to recruit fibroblasts.

### Sequential fibroblast activation and EC migration led by LSCs

**Figure 6:**
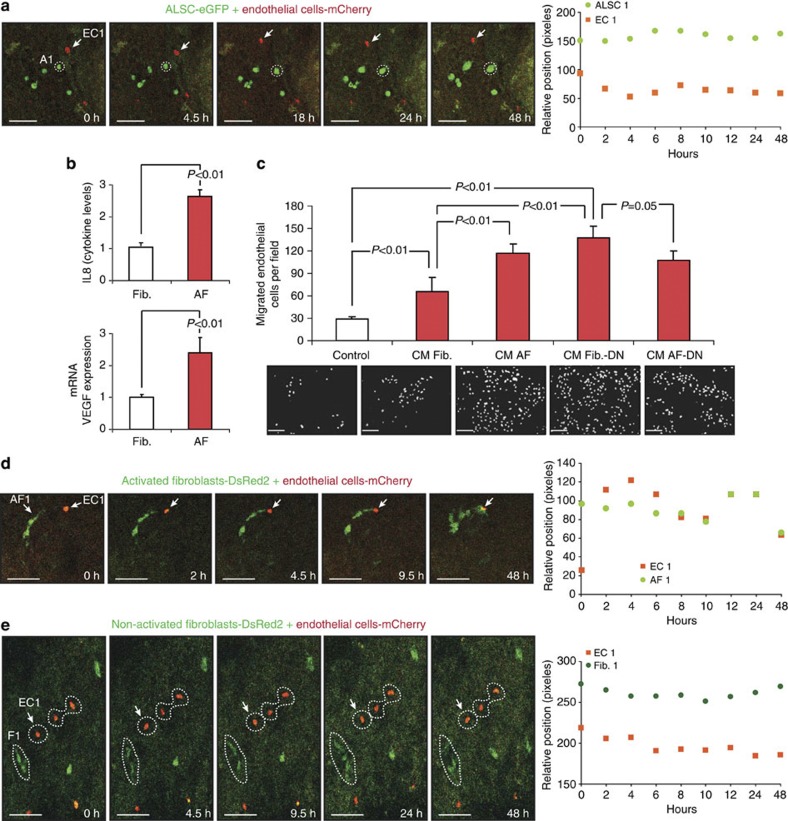
Recruitment of endothelial cells by previously LSCs-activated fibroblasts. (**a**) Time-lapse tracing of ALSCs (green) and endothelial cells (red) injected in lung explants. The graph shows the relative positioning of ALSCs and endothelial cells. Scale bar, 200 μm. (**b**) Release of IL8 protein and VEGF mRNA expression are promoted in fibroblasts co-cultured with LSCs (AF). All results are the mean of three triplicate experiments±s.e.m. *P*<0.01 values were defined as statistically significant, as analysed by one-way ANOVA. (**c**) Promotion of endothelial cell migration by conditioned medium (CM) from AF, Fib.-DN or AF-DN. A representative experiment (±s.e.m.) of five independent triplicates is shown. *P*<0.01 values were defined as statistically significant, as analysed by one-way ANOVA. Scale bar, 200 μm. (**d**) Time-lapse images show the recruitment of endothelial cells (red) by activated fibroblasts (green) when injected in lung explants. (**e**) Non-activated fibroblasts (green) are unable to induce endothelial cell (red) recruitment in lung explants. The graphs in **d** and **e** show the relative position of endothelial cells and fibroblasts. Scale bar, 200 μm.

Previous studies have suggested that stem cells can have the potential to cross-talk with vascular cells^9^. After showing the paracrine activation of fibroblasts by LSCs and taking into account the results from kidney engraftments, the potential direct recruitment of endothelial cells (EC) by ALSCs was investigated. Surprisingly and unlike fibroblasts, ALSCs fail to recruit endothelial cells in lung explants and to migrate *in vitro* (Fig. 6a and Supplementary Fig. 5A).

Interestingly, increased expression and release of the angiogenic factors IL8 and VEGF in AFs was observed (Fig. 4g, Fig. 6b and Supplementary Fig. 5B), suggesting a putative role of these molecules in endothelial cell recruitment. This expression was independent of p38α activity, as Fib.-DN also showed increased expression of angiogenic factors (Fig. 4g and Supplementary Fig. 5C) and responded to ALSC induction (Fig. 4g and Supplementary Fig. 5D).

Inhibition of p38α activity may result in a constitutive activation of the JNK pathway^7^. Indeed, Fib.-DN had a constitutively activated JNK/AP-1 pathway, which was also present in AFs following 12 h of co-culture (Supplementary Fig. 5E). AP-1 has been shown to regulate the expression of both VEGF and IL8 (refs 23, 24, 25, 26). The direct role of AP-1 was confirmed, as inhibition of JNK activity by a small molecule inhibitor (SP600125) prevented the induction of both IL8 and VEGF expression in AFs by ALSCs (Supplementary Fig. 5F). Consequently, CM from AFs, Fib.-DN or AF-DN, induced a higher migration of endothelial cells than CM from WT fibroblasts (Fig. 6c), independently of proliferation (Supplementary Fig. 5G). Importantly and consistent with the above results, only AFs (Fig. 6d and Supplementary Fig. 5H) but not regular fibroblasts (Fig. 6e and Supplementary Fig. 5I) were able to recruit endothelial cells in lung explants.

Therefore, endothelial migration and recruitment is mediated by angiogenic signals, induced by the JNK/AP-1 pathway in AFs, but not by direct paracrine signals from ALSCs.

### SDF-1 induces TNFα and angiogenic factors in fibroblasts

We have shown that there are two signalling pathways (p38α and JNK/AP-1) activated in fibroblasts only when they are co-cultured with LSCs, suggesting that those fibroblasts are also regulated by paracrine factors from LSCs. We hypothesized that SDF-1 could be the factor responsible to activate both pathways. To test this hypothesis, either shControl LSCs or LSCs lacking SDF-1 (LSC-KD) were co-cultured with fibroblasts. We found that fibroblast activation was dependent on SDF-1. LSCs lacking SDF-1 did not induce p38α at 6 h (Supplementary Fig. 6A), nor JNK/AP-1 activation at 12 h (Supplementary Fig. 6B), correlating with the reduced expression of TNFα and angiogenic factors (IL8, VEGF), respectively (Supplementary Fig. 6C,D). However, activation of both pathways and expression of those cytokines could be rescued by addition of recombinant SDF-1 (Supplementary Fig. 6E).

Moreover, we observed that low levels of SDF-1 induced early expression of TNFα, but only high levels could induce the expression of VEGF and IL8 in fibroblasts (Supplementary Fig. 6F). Thus, SDF-1 promoted early activation of p38α, which correlates with TNFα expression, and later JNK/AP-1 activation that correlated with IL8 and VEGF expression in AFs.

Interestingly, real-time qPCR analysis showed that TNFα and VEGF were upregulated in kidney engraftments in comparison with mouse fibroblasts, giving the former cytokines an *in vivo* role in the engrafting and microenvironment process initiated by LSCs (Supplementary Fig. 6G).

Finally and consistent with the above results, we confirmed the essential cross-talk between LSCs and fibroblasts to regulate endothelial cell migration. We found that CM from fibroblasts co-cultured with SDF-1-deficient stem cells (AF+LSC-KD) failed to enhance endothelial migration (Supplementary Fig. 6H), but it did not affect their proliferation (Fig. 6i).

Altogether, these findings are consistent with the results from kidney grafts, in which LSCs lacking SDF-1 failed to recruit stromal cells as a result of the disruption of the TNFα-TGFβ-p38α-SDF-1 network.

**Figure 7:**
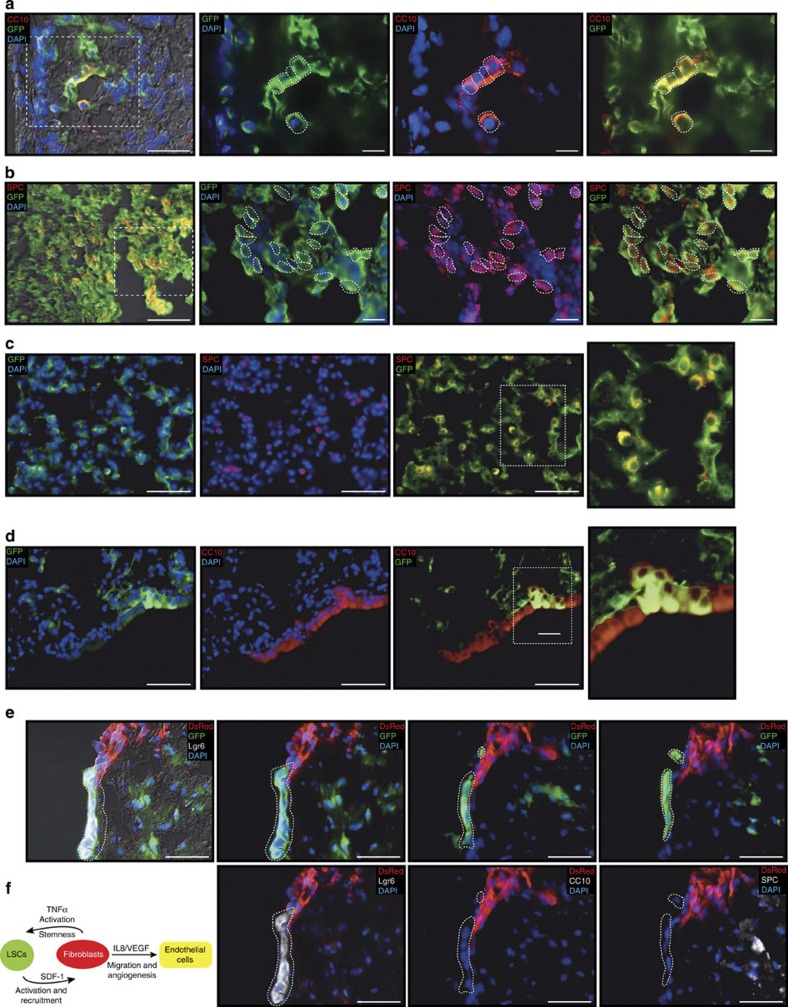
Analysis of LSC differentiation controlled by stromal fibroblasts. In kidney capsule engraftments, LSC-KD cells (GFP-labelled) overexpressing a shSDF-1 resistant mRNA can differentiate either in (**a**) bronchiolar Clara cells (CC10^+^) or in (**b**) alveolar AT2 cells (SPC^+^) (see also Fig. 3b). (**c**,**d**) *Ex vivo* lung explants showing the ability of LSCs (GFP-labelled) to differentiate in (**c**) alveolar AT2 cells (SPC^+^) and (**d**) in bronchiolar Clara (CC10^+^) cells. (**e**) Immunofluorescence images of serial sections of lung explants. LSCs (GFP-labelled), which are in contact with fibroblasts (DsRed-labelled), express the stem cell marker Lgr6 but not the lung differentiation markers SPC or CC10. (**f**) A model that summarizes the paracrine cross-talk between stromal cells and LSCs. Scale bar, 50 μm.

The results allow us to delineate a model that can explain how lung stem cells promote the formation of their own environment, a necessary contributor to generating a lung epithelium. LSCs engage in cross-talk with fibroblasts. Basal levels of SDF-1 from LSCs can induce both the recruitment and priming of fibroblasts. ‘Low activated’ fibroblasts release TNFα, leading to subsequent activation of a TGFβ/p38α autocrine loop in LSCs (ALSCs) and further promotion of SDF-1 expression. Higher levels of SDF-1 induce a second and enhanced expression of TNFα (through p38α) in fibroblasts (AFs). High SDF-1 levels are also able to induce expression of angiogenic factors (through the JNK/AP-1 pathway) in fibroblasts. IL8 and VEGF released by ‘highly activated’ fibroblasts then recruit endothelial cells, thus promoting angiogenesis (Fig. 7f).

### Fibroblast-dependent control of stem cell differentiation

We have begun to identify the paracrine signals required to maintain a functional cross-talk between stem and stromal cells. Indeed, this cross-talk is essential because, in kidney engraftments, rescued LSC-KD cells by SDF-1 overexpression are able not only to recruit fibroblasts and endothelial cells to the graft (see Fig. 3c) but also to differentiate into bronchiolar (Fig. 7a) and alveolar (Fig. 7b) cells. These results suggest that SDF-1 released by LSCs is essential to recruit stromal cells and also to allow engraftment and—consequently—differentiation of LSCs into the kidney capsule. Why the stromal cells are necessary to develop a LSC microenvironment is still unknown. We have previously reported that grafts from single LSCs injections harboured small pools of Lgr6^+^ undifferentiated cells, which retain self-renewal ability^8^. We hypothesized that stromal cells are required to maintain this potential.

To test this hypothesis, we injected LSCs and fibroblasts in human lung explants to allow cell recruitment. After 10 days we analyzed the expression of several differentiation markers in serial sections by immunofluorescent microscopy. Consistent with our hypothesis, now we provided unambiguous evidence that LSCs located away from fibroblasts are allowed to start lineage commitment as they engraft into the epithelial tissue differentiating into AT2 (SPC+), Clara (CC10+) or AT1 (AQ5+) cells (Fig. 7c, d, and Supplementary Fig. 7A,B). In contrast, LSCs closer to fibroblasts remain in an undifferentiated state, expressing the stem cell marker Lgr6 but not the lung differentiation markers SPC (Surfactant protein C), CC10 (Clara cell 10) or AQ5 (Aquaporin 5) (Fig. 7e).

These findings suggest that LSCs induce the recruitment of stromal cells to create a functional microenvironment to ensure self-renewal capacity. Once this capability is ensured, LSCs can start the differentiation process towards bronchiolar or alveolar lineage commitment.

## Discussion

Tissue homeostasis is maintained by the integration of a number of physiological signals derived from cellular and matrix interactions, and autocrine, paracrine and hormonal signals. Proper knowledge of the signals and the cell types involved in the response to physiological and pathological insults is essential to understand the maintenance of a healthy and functional organ.

Many reports highlight the connection of the endodermal-mesenchymal cross-talk and alveolarization during development of the mouse embryonic lung^27^,^28^,^29^. In the adult mouse lung, the role of angiogenic signals in lung alveolarization^12^ and the role of lung fibroblasts in lung epithelial differentiation *in vitro*^30^ have also been established. However, a better understanding of the signals released by specific cell types, the intracellular mediators, and the balance of their cross-talk is still necessary.

Here we establish the importance of the paracrine cross-talk between an enriched LSC population and fibroblasts in the recruitment of stromal cells and in the posterior maintenance of the stem cell potential of LSCs. LSCs closer to fibroblasts remain in an undifferentiated state, ensuring self-renewal capacity. Once this ability is ensured, LSCs can leave their microenvironment and respond to differentiation signals leading to lineage commitment.

In this manuscript, we have revealed the potential of serially propagated, clonally derived enriched Lgr6+/E-Cad+ (LSC) populations to recruit stromal cells, which influence the potential of LSC to maintain their status (that is, stemness) or differentiate into mature bronchioalveolar epithelial cell lineages. However, it is important to note that these serially propagated Lgr6+/E-Cad+ cells are heterogeneous. While their ability to generate bronchioalveolar epithelium when injected under the kidney capsule, and to differentiate into alveolar or bronchiolar cells in *ex vivo* lung explants, is consistent with the enrichment of LSC activity, it does not prove that the inoculum comprises individual cells with homogeneous functional attributes. Although the assays used are considered the best available to test self-renewal and differentiation potential of human cells, we acknowledge their limitations. Consequently, although we believe our data are consistent with the interpretation that Lrg6+ (LSCs) are the key cells that mediate the cross-talk with fibroblasts, we cannot exclude the possibility that other Lrg6+ cells with diminished or no regenerative potential also participate in the cross-talk with fibroblasts to maintain LSC status in a functional microenvironment.

We have provided new elements to delineate a functional and temporal hierarchy, for stem and stromal cell activation in the human adult lung. In this circuit, LSCs released basal levels of SDF-1 that moderately activated lung fibroblasts leading to the production of paracrine signals, and in particular TNFα. This cytokine in a positive feedback loop activates LSCs (ALSCs), inducing a TGFβ autocrine loop that enhances the expression of SDF-1. High SDF-1 levels promoted the recruitment and further activation of fibroblasts (AFs). ALSCs exploited AFs for endothelial cell recruitment. Only AFs primed by high SDF-1 levels, expressed the angiogenic factors VEGF and IL8, needed for endothelial chemoattraction.

Intracellularly, the p38α MAPK pathway is key to maintain the auto-regulation of this circuitry. In fibroblasts, p38α is involved in responding to SDF-1 and induce cytokine expression, including TNFα. Lack of p38α MAPK signal resulted in deficient cytokine release and abrogation of the feedback loop to activate LSCs. Furthermore, p38α signal in ALSCs mediated TGFβ-induced SDF-1 expression. The secretion of this chemoattractant is essential for LSCs potential to promote their own niche. This property of LSCs is crucial to generate a lung-like epithelium in the kidney capsule, and lack of p38α or SDF-1 avoids engraftment. *In vitro* and *in vivo* rescue by SDF-1 overexpression or addition of the recombinant protein confirmed that it is essential in the circuit. The role of p38α guaranteeing the differentiation potential relies in the previous establishment of a niche, but this is not sufficient, as shown by overexpression of SDF-1, which cannot rescue the lack of p38α in promoting LSCs bronchioalveolar differentiation.

Paracrine cross-talk and activation of stromal fibroblasts are essential for lung stem cells to initiate and maintain a functional tissue. Many efforts have been made in the past years trying to understand the signals controlling lung homeostasis and their involvement in pathological processes. We have begun to define a circuitry of intracellular and extracellular components involved in the setting and maintenance of the system^8^. We now provide new targets that include soluble molecules and intracellular pathways that can be targeted in specific cell types to modify or restore proper regeneration of the human lung alveolar epithelium, or to target metastatic lung adenocarcinoma cells.

## Methods

### Cell culture and isolation of human lung cells

Human umbilical vein endothelial cells (HUVEC) were purchased from Lonza and cultured in EGM BulletKit™ medium (Lonza, #CC-3124). Fibroblasts, obtained from human lung tissue, were negatively sorted for CD45^−^/CD31^−^/E-Cadherin^−^/Lgr6^−^ to avoid hematopoietic, endothelial, epithelial and stem cell contamination. Negative sorted cells were then put in culture in DMEM medium containing 10% fetal bovine serum (FBS). An aliquot of these cells was fixed, permeabilized and labelled with antibodies to Vimentin, confirming that 99% of the cells were Vimentin^+^.

Human lung stem cells were isolated as describe previously^8^. Human lung tissue was obtained from patients undergoing lung resection at Papworth Hospital, UK. All subjects gave informed consent. Normal lung specimens were finely minced, resuspended in DMEM containing a mix of collagenase (0.5–3 mg ml^−1^, Whorthington)/dispase (1 mg ml^−1^, Invitrogen) and incubated for 30–45 min at 37 °C in a shaking incubator. The suspension was spin for 5 min at 1,200 r.p.m. and the supernatant removed. The pellet was resuspended in fresh DMEM containing 0.1 mg ml^−1^ DNase (optional) and incubated for further 5–10 min. The suspension was washed with PBS, filtered through cell strainers (100 μm and 70 μm, BD Falcon) and treated with red blood cell lysis buffer (Roche Applied Science). Following further filtration (40 μm mesh) and centrifugation (5 min at 1,200 r.p.m.), the isolated cells were cultured in RHB-A medium containing 2% FCS, with additional insulin (5 μg ml^−1^, Pepro Tech), EGF (10 ng ml^−1^, Pepro Tech) and FGF2 (20 ng ml^−1^, Pepro Tech) for 2 days. Cells were then sorted in a flow cytometer. Cells were first negatively sorted for Lyn- (CD45^−^/CD73^−^/CD31^−^/CD34^−^/CD33^−^) to avoid mesenchymal, hematopoietic and endothelial cell contamination. Negative sorted cells were then double sorted for E-Cadherin^+^/Lgr6^+^ and then put in culture or used for *in vivo* assays. Cells in culture were maintained in serum-free medium containing EGF and FGF2 (37 °C in a 7% humidified CO_2_ incubator). All the experiments were performed with lung stem cells derived from single cell clones as we previously described^8^. Single cells were seeded into 96-well plates by limited dilutions and maintained in stem cell restriction medium RHB-A containing EGF and FGF2. After 14 days, the number of wells with colonies was counted. Every assay was repeated four times.

### Tracking cells in Bleomycin-treated lung explants

Human or adult mouse lungs were cultured as slices (600–900 μm thickness) and exposed to bleomycin (5 U kg^−1^ in 100 μl PBS) for 2–3 days *in vitro*. As a control, lung slides were incubated for 2–3 days in N2B27 medium containing 2–5% FBS. After injury, EGFP-labelled LSCs, DsRed2-labelled fibroblasts or mCherry-labelled endothelial cells were microinjected into the lungs and cultured for 7–10 days.

For tracking of LSCs and fibroblasts, EGFP-labelled LSCs (1 × 10^6^ cells) and DsRed2-labelled fibroblasts (3 × 10^5^ cells) were resuspended in 1 ml of PBS and then microinjected (50–100 μl) into the lung slides and cultured for 7–10 days in N2B27 medium containing 2–5% FBS.

For tracking of LSCs and endothelial cells, EGFP-labelled LSCs (3 × 10^5^ cells) and mCherry-labelled endothelial cells (3 × 10^5^ cells) were resuspended in 1 ml of EGM medium (containing 5% FBS) and then microinjected (50–100 μl) into the lung slides and cultured in the same medium.

For tracking of fibroblasts and endothelial cells, DsRed2-labelled fibroblasts (activated or non-activated) (3 × 10^5^ cells) and mCherry-labelled endothelial cells (3 × 10^5^ cells) were resuspended in 1 ml of EGM medium (containing 5% FBS) and then microinjected (50–100 μl) into the lung slides and cultured in the same medium.

For cell tracking a Leica Confocal microscope was used. Pictures of selected regions were taken every day over a period of 7 days. Manual cell tracking was performed for each cellular type that stayed within the region of analysis with ImageJ software using the MtrackJ plugin. For live imaging a Leica Confocal microscope was used. Pictures of selected regions were taken every 20–30 min over a period of 48 h.

For rescue experiments, LSCs knockdown for SDF-1 were co-injected with recombinant human SDF-1α/CXCL12 (200 ng ml^−1^) (R&D Systems, #350-NS-010) and matrigel. Alternatively, LSCs knockdown for SDF-1 were transduced with viruses expressing an shSDF-1 resistant mRNA. For inhibition experiments, 1 μg ml^−1^ of anti-TNFα (R&D Systems, #MAB4101) was added to each well. Lung slides were fixed for 1–2 h with 4% paraformaldehyde at room temperature. Tissues were then processed for cryosectioning.

### Co-culture assay and preparation of conditioned medium

For co-culture experiments, cells were cultured at a seeding ratio of 1:3 (LSCs:fibroblasts) in Boyden chambers (BD Falcon, #353091). A total of 200,000 fibroblasts were seeded in six-well plates 24 h before the start of the assay. Fibroblasts were then washed with PBS and 70,000 LSCs were added into each of the upper chambers in triplicates. Human lung stem cells were co-cultured with fibroblasts in inserts having a 3.0 μm porous membrane in serum-free DMEM medium. Thus, the two cell types shared the same culture medium but did not physically contact each other. After 48 h of co-culture, the conditioned medium generated by each cellular type was collected and filtered for its analysis or used it for *in vitro* assays. LSCs and fibroblasts were processed for western blotting or quantitative RT–PCR. For antibody experiments, 1 μg ml^−1^ of anti-TNFα (R&D Systems, #MAB4101) or anti-TGFβ (R&D Systems) was added to each well of the lower or the upper chamber in triplicates, respectively. For inhibition experiments, fibroblasts were incubated with 5–10 μM of JNK inhibitor SP600125 (Sigma), and the assay was allowed to proceed for 24 h.

For rescue experiments, after 24 h of co-culture, recombinant human TGFβ1 (10 ng ml^−1^) (Cell Signalling, #8915) or recombinant human TNFα (100 ng ml^−1^) (Cell Signaling, #8902) was added to the upper chamber and incubated for 6 h or 12 h, respectively.

### Cytokine assays

Cytokine Array Panel A (R&D Systems, #ARY006) was used to determine the cytokines present in the medium of co-cultured LSCs and fibroblasts. After 48 h of co-culture, the conditioned medium was transferred to the array, and the analysis was done according to the manufacturer’s instructions. The signals were detected and quantified using the Odyssey Infrared Imaging System (Li-Cor, Biosciences).

### Flow cytometry

Single-cell flow cytometry was performed (Fortesa/BD Biosciences) following fixation and incubation with Muc5AC (1/1,000, Abcam, ab3649), CGRP (1/1,000, Abcam, ab 81887), SPC (1/500, Santa Cruz, sc-7706), CC10 (1/1,000, Santa Cruz, sc-25555) and Vimentin (1/1,000, BD Pharmigen, #550513) primary antibodies, using a BD-Fortessa machine. Secondary antibody (1/5,000, Alexa Fluor 488 and/or 1/5,000, Alexa Fluor 555, 1/5,000, Alexa Fluor 647 secondary antibodies, Invitrogen) incubation took place for 30 min at RT. Data were processed using FlowJo.

### Cellular treatments

To analyse SDF-1 expression, LSCs were treated with recombinant human TGFβ1 (10 ng/ml) (Cell Signaling, #8915) for 6 h or with recombinant human TNFα (100 ng/ml) (Cell Signaling, #8902) for 12 h. For western blotting, LSCs were treated with recombinant human TNFα (10 ng ml^−1^) for 15 min. For inhibition experiments, LSCs were pre-incubated with a JNK inhibitor SP600125 (5–50 μM) 60 min before TNFα treatment.

For JNK and p38αMAPK activation, fibroblasts were seeded in six-well plates 24 h before the start of the assay. Then, the medium was changed to DMEM containing 2% FBS. After 6 h the medium was replaced with DMEM containing 0.5% FBS and recombinant human SDF-1α/CXCL12 (200 ng ml^−1^) (R&D Systems, #350-NS-010) was added to each well. The same samples were processed for real-time qPCR to measure TNFα, VEGF and IL8 expression.

### Fibroblasts and HUVEC migration assay

The *in vitro* migration assays were carried out in Boyden chambers of 8.0 μm porous size (BD Falcon, #353097) using the protocol described by ref. 31. A total of 100,000 HUVEC cells or fibroblasts were resuspended in 1 ml of 0.2 or 0.5% FBS DMEM medium, respectively. A 0.5 ml of the re-suspension was then added into each of the upper chambers in triplicates. Cells were then stimulated with 0.8–1 ml conditioned medium added to the lower chamber. After 16–20 h of incubation, the non-migrated cells on the upper side of the chamber membranes were removed. The migrated cells to the basal side of the chamber membranes were fixed with methanol for 10 min at room temperature. Migrated cells were visualized with DAPI (4′,6-diamidino-2-phenylindole) and counted in 5–10 fields per membrane using ImageJ.

For rescue experiments, conditioned medium was supplemented with recombinant human SDF-1α/CXCL12 (30 ng ml^−1^) (R&D Systems, #350-NS-010).

For fibroblasts recruitment assays that required preincubation with antibodies, conditioned medium was preincubated with 5 μg ml^−1^ of anti-human SDF-1/CXCL12 antibody (R&D Systems, #AF-310-NA) or control IgG antibody for 60 min and then added to each well.

### Fibroblast and HUVEC proliferation

A total of 30,000 fibroblasts or 25,000 HUVEC cells were seeded in triplicate into 24-well plates 24 h before the start of the proliferation assay. Fibroblasts or HUVEC cells were then washed with PBS and 1 ml conditioned medium was added to each well. After 48 h, the conditioned medium was replaced with another 1 ml of conditioned medium. Every 24 h, the cells were trypsinized and counted using a haemocytometer.

### shRNA-mediated messenger RNA knockdown

A commercial shRNA (Sigma, NM_000609.4-247s21c1) was used to knockdown SDF-1/CXCL12 expression: ‘5′-CCGGCAAACTGTGCCCTTCAGATTGCTCGAGCAATCTGAAGGGCACAGTTTGTTTTTG-3′’ (underline letters indicate the target region). Lentiviral particles were produced by co-transfecting 293T cells with pLKO.1-puro, pCMV Δ8.9 and VSV-G. Culture supernatants were collected 48 h after transfection and filtered through 0.45 μM membranes. The supernantans were concentrated by centrifugation at 28,000 r.p.m. (2 h at 4 °C) and resuspended in BSA 1%. LSCs were then transduced with lentiviruses for 16 h in the presence of 8 mg ml^−1^ polybrene (hexadimethrine bromide, Sigma).

### Retroviral vector expressing RNAi-resistant SDF-1

A commercial pBabe-SDF-1α (Addgene, ♯12270) vector was used to generate an RNAi-resistant SDF-1. Introducing six silent mutations within shRNA target region generated the RNAi-resistant SDF-1. The target sequence of SDF-1 mRNA was 5′-CAAACTGTGCCCTTCAGATTG-3′, and the target sequence of RNAi-resistant SDF-1 mRNA was 5′-CCA AA**T** TG**C** GC**G** CT**G** CA**A** AT**A** G-3′ (bold letters indicate the silent mutations).

### Western blotting

Cells were lysed in lysis buffer (50 mM Tris-HCl pH 7.5, 150 mM NaCl, 1% (v/v) NP-40, 5 mM EDTA pH 8.0, 5 mM EGTA pH 8.0, 20 mM NaF, 0.1 μM PMSF, 0.1 μM NaVO_3_, plus complete protease inhibitor cocktail (Roche)) and cellular lysates were separated by SDS–PAGE and transferred to PVDF membranes. The following antibodies were used for protein detection: p38α MAPK (1/1,000, Cell Signaling, #9228), phospho-p38 (Thr180/Tyr182, 1/1,000, Cell Signaling, #9215), phospho-c-Jun (Ser63, 1/1,000, Cell Signaling, #9161), c-Jun (1/1,000, Cell Signaling, #L70B11), phospho-JNK (Thr183/Tyr185, 1/1,000, Cell Signaling, #9255), JNK (1/1,000, Cell Signalling, #9258), phospho-Smad2 (Ser465/Ser467, 1/1,000, Millipore, #AB3849), Smad2 (1/1,000, Cell Signalling, #3103), human CXCL12/SDF-1 antibody (1/1,000, R&D Systems, #AF-310-NA), tubulin (Sigma). For detection, we used Alexa Fluor 680- (1/5,000, Molecular Probes) or Li-Cor IRDye 800- (Rockland) labelled antibodies with the Odyssey Infrared Imaging System (Li-Cor).

### Total RNA isolation and quantitative RT-PCR

Total RNA was extracted using Trizol (Invitrogen) and treated with RNase-free DNAse I (Promega). One microgram RNA was reverse transcribed (Biorad), according to the manufacturer’s instructions. Quantitative real-time PCR (qPCR) was used to determine the expression levels of the different genes using specific primer pairs (Eppendorf, Realplex^2^) (Supplementary Table 5). Reaction conditions for amplification were as follows: first step of 95 °C 20 s, then 40 cycles of three-step 95 °C 3 s, 60 °C 30 s and 68 °C 20 s with 2 μl of cDNA per reaction in 10 μl SYBR Green PCR Master Mix (Applied Biosystems). Specificity of PCR products was tested by dissociation curves. Threshold cycles of primer probes were normalized to a housekeeping gene (GAPDH or β-actin) and relative values calculated.

### Kidney capsule engraftments

All mouse experiments were performed according to UK Home Office Regulations, with the approval of the Ethics Committee at Cambridge University. CD-1 nude mice (Charles River) were maintained under standard pathogen-free conditions. Six- to eight-week-old male mice were anaesthetized with isoflurane (0.5–2%). LSCs WT or LSCs overexpressing a shRNA to knockdown SDF-1/CXCL12 were disassociated with accutase to generate a single-cell suspension (0.5–1 × 10^5^ cells in 10 μl PBS), and this suspension was injected under the kidney capsule. Mice were killed 2, 3 and 4 weeks later and the kidneys were collected to examine *in vivo* differentiation of the injected cells or to analyse the recruitment of stromal cells. Grafts were removed and prepared for immunofluorescent microscopy.

For rescue experiments, LSCs overexpressing a shRNA to knockdown SDF-1/CXCL12 (LSC-KD) were co-injected with recombinant human SDF-1α/CXCL12 (40–50 ng μl^−1^) (R&D Systems, #350-NS-010) and matrigel under the kidney capsule. Alternatively, LSC-KD cells expressing a shSDF-1 resistant mRNA were injected under the renal capsule. Grafts were removed after 2 and 4 weeks and prepared for immunofluorescent microscopy.

### Histology and immunostaining

Kidney capsule grafts or lung slides were fixed with 4% paraformaldehyde and embedded in OCT. Samples were sectioned at 6–12 μm sections. The following primary antibodies were used: anti-human CC10 (1/1,000, Santa Cruz, sc-365992), anti-human SPC (1/1,000, Santa Cruz, sc-7705), anti-human AQP5 (1/500, Santa Cruz, sc-9890), anti-human LGR6 (1/1,000, Santa Cruz, SC-48236), anti-human SDF-1 (1/500, Santa Cruz, sc-6193), anti-human CXCR4 (1/500, Santa Cruz, sc-9046), anti-GFP (1/1,000, Abcam, ab-13970), anti-RFP (1/1,000, Abcam, ab-62341), anti-CD73 (1/1,000, Abcam, ab-54217), anti-mouse Vimentin (1/1,000, BD Pharmigen, #550513), anti-human Nuclei antibody (1/1,000, Millipore, MAB1281). Sections were incubated in blocking buffer (PBS, 4% donkey serum, 1% Triton) for 1 h at room temperature. Primary antibodies were incubated overnight at 4 °C. Sections were rinsed three times in PBS and incubated with secondary antibodies diluted at 1:1,000 for 1 h at room temperature. Slides were mounted in Vectashield mounting media with DAPI (4′,6-diamidino-2-phenylindole).

## Author contributions

E.J.R. performed and designed experiments, analysed results and wrote the manuscript. F.O-.W. performed and designed experiments. J-.J.V. designed experiments, analysed results and wrote the manuscript.

## Additional information

**How to cite this article:** Ruiz, E. J. *et al.* A paracrine network regulates the cross-talk between human lung stem cells and the stroma. *Nat. Commun.* 5:3175 doi: 10.1038/ncomms4175 (2014).

## Supplementary Material

Supplementary Information Supplementary Figures 1-8 and Supplementary Tables 1-5
