# Supplementary material for: A paracrine network regulates the cross-talk between human lung stem cells and the stroma
Source: Nat Commun. 2014 Jan 16;5:3175. doi: 10.1038/ncomms4175 (PMC3905720; doi:10.1038/ncomms4175)
Supplement: Supplementary Information — Supplementary Figures 1-8 and Supplementary Tables 1-5 [file ncomms4175-s1.pdf]

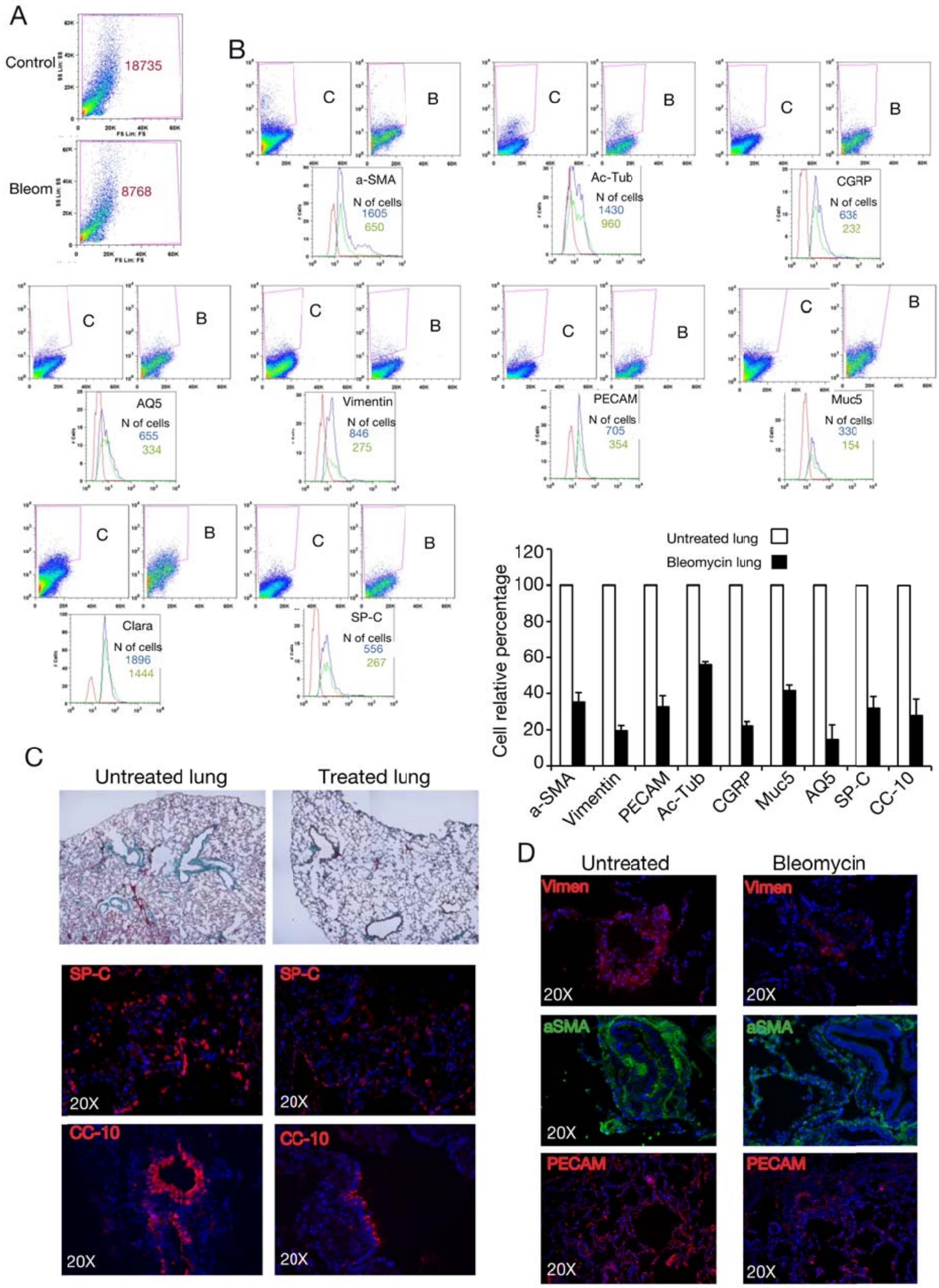
A
Control
18735
Bleom
8768
B
C
B
a-SMA
N of cells
1605
650
Ac-Tub
N of cells
1430
960
CGRP
N of cells
636
232
AQ5
N of cells
655
334
Vimentin
N of cells
846
275
PECAM
N of cells
705
354
Muc5
N of cells
330
154
Clara
N of cells
1896
1444
SP-C
N of cells
556
267
Untreated lung
Bleomycin lung
Cell relative percentage
0
20
40
60
80
100
120
a-SMA
Vimentin
PECAM
Ac-Tub
CGRP
Muc5
AQ5
SP-C
CC-10
C
Untreated lung
Treated lung
SP-C
CC-10
20X
D
Untreated
Bleomycin
Vimen
aSMA
PECAM
20X

Ruiz et al.,

Supplementary Figure 1. Bleomycin induces general cell death in lung explants. (A) Flow cytometry plot shows the loss of cell numbers in lung explants treated with bleomycin for 3 days. 6 explants per lung with the same size (10mg) were obtained from 5 different lungs and culture for the experiment (15 control, 15 bleomycin). (B) Lung explants were dissociated and single cells fixed and permeablized for flow cytometry analysis. Flow cytometry shows the plots for the different markers in untreated (C) or treated (B) lung explants and the histograms depict the positive cells for each cell type and the number of untreated (blue) and bleomycin treated (green) explants (negative control in red). The graph shows the relative percentage of mesenchymal (a-SMA, vimentin), endothelial (PECAM), bronchiolar (Ac-Tubulin, Muc5, Clara), neuroendocrine (CGRP) or Alveolar (SP-C, AQ5) cells obtained by flow cytometry analysis, taken as the normalized (to untreated) average differential cell number between untreated or bleomycin-treated cells, from the comparison between explants from each lung. (C) Masson-Trichome staining (upper panels), and immunofluorescence images showing expression of lung specific markers in untreated and bleomycin-treated of lung explants *in vitro.* (D) Immunofluorescence of non-treated or bleomycin-treated explants expressing mesenchymal and endothelial markers.

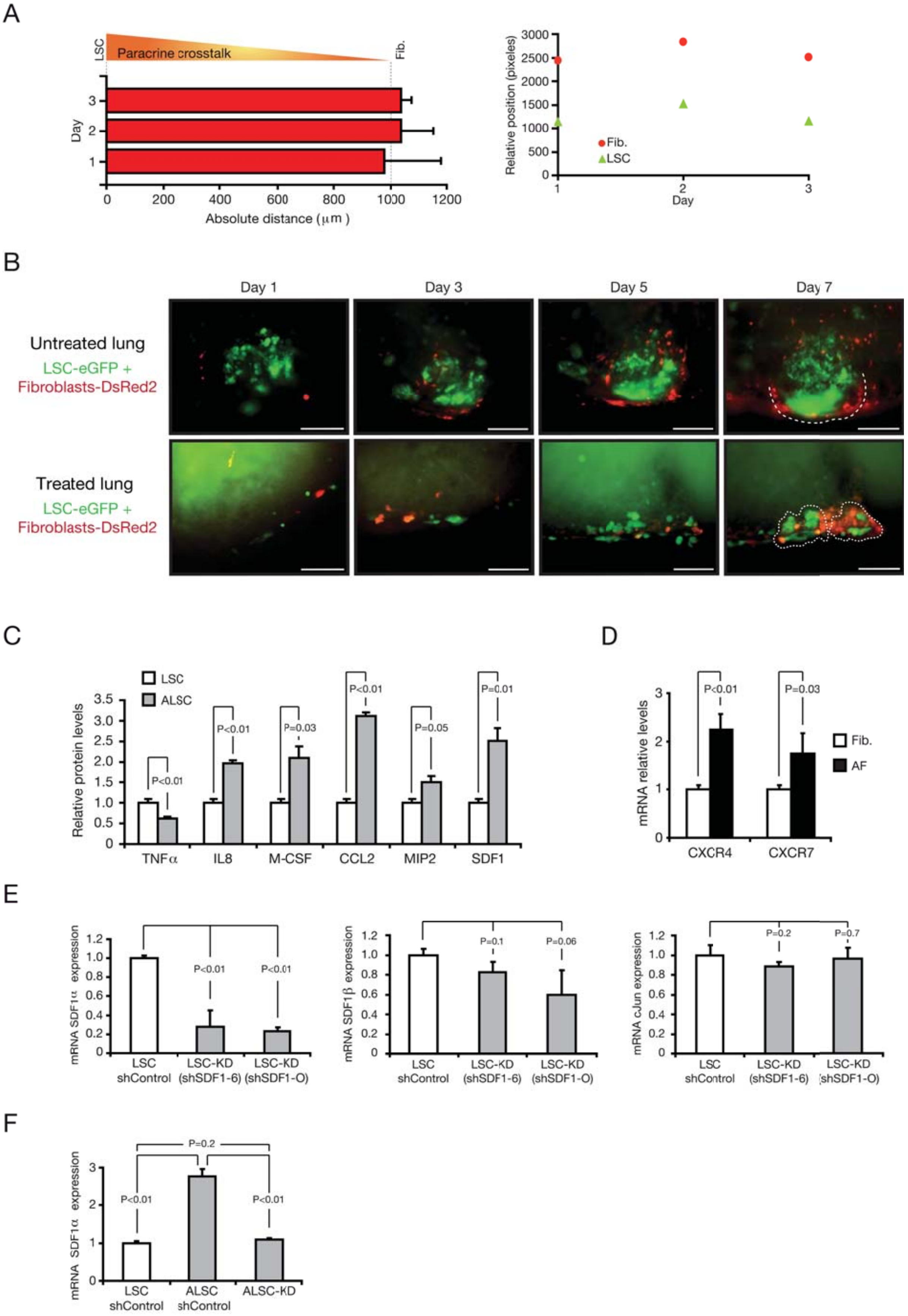

A
LSC
Paracrine crosstalk
Fib.
Day
Absolute distance (μm)
Relative position (pixeles)
Fib.
LSC
Day
B
Day 1
Day 3
Day 5
Day 7
Untreated lung
LSC-eGFP +
Fibroblasts-DsRed2
Treated lung
LSC-eGFP +
Fibroblasts-DsRed2
C
LSC
ALSC
Relative protein levels
P<0.01
P<0.01
P=0.03
P<0.01
P=0.05
P=0.01
TNFα
IL8
M-CSF
CCL2
MIP2
SDF1
D
mRNA relative levels
P<0.01
P=0.03
Fib.
AF
CXCR4
CXCR7
E
mRNA SDF1α expression
P<0.01
P<0.01
LSC shControl
LSC-KD (shSDF1-6)
LSC-KD (shSDF1-O)
mRNA SDF1β expression
P=0.1
P=0.06
mRNA cJun expression
P=0.2
P=0.7
F
mRNA SDF1α expression
P=0.2
P<0.01
P<0.01
LSC shControl
ALSC shControl
ALSC-KD

Supplementary Figure 2. Ex vivo analysis of LSC-fibroblasts crosstalk and cytokine expression and generation of LSCs lacking SDF-1. (A) The graphs show the distance-dependent recruitment of fibroblasts from LSCs injected in lung explants. The data are shown as the mean ± S.E.M. of 3 triplicate experiments. (B) Images showing time-lapse recruitment of fibroblasts (red) by LSCs (green) injected in untreated or bleomycin-treated lung explants. Scale bars: 200μm. **(**C) Relative protein levels of various cytokines released into the medium by LSCs or ALSCs. (D) Comparative mRNA expression of SDF-1 receptors in fibroblasts or AFs. (E) Inhibition of SDF-1 expression in LSCs by two different short hairpin RNAs that are stably expressed from a lentiviral vector. shSDFs expressed in LSCs specifically knockdown SDF-1α expression (left panel) but they do not affect the levels of the other isoform, SDF-1β (middle panel). shSDFs do not affect cJun expression in LSCs (right panel). (F) Short hairpin against SDF-1 but not shControl, prevented SDF-1 expression in activated LSCs (ALSC-KD). All results (Fig. C-F) are the mean ± S.E.M. of 4-5 triplicate experiments. P<0.01 values were defined as statistically significant, as analysed by One-way ANOVA.

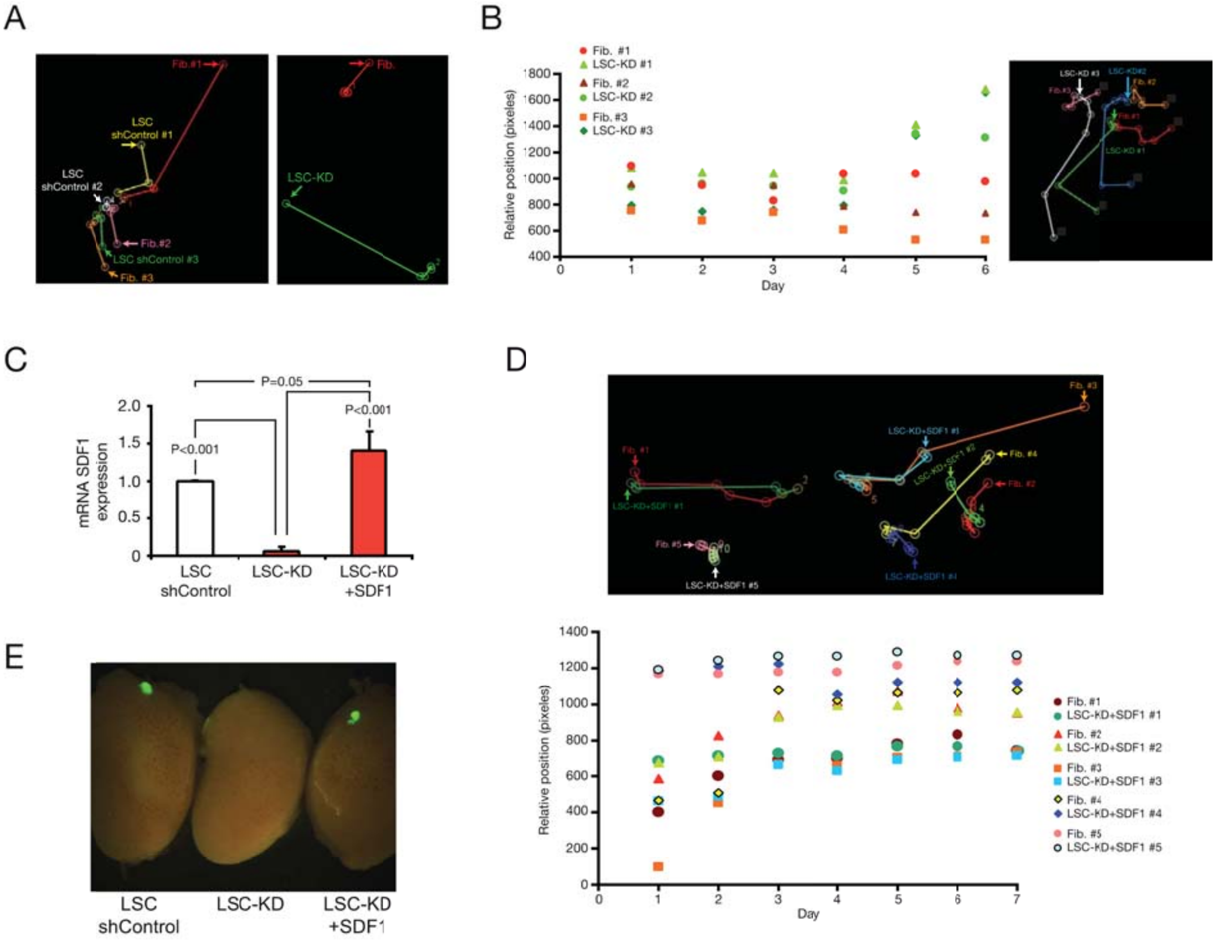

F

	LSC shControl	LSC-KD	LSC-KD+ SDF1+matrigel
No. Engrafments (%)	4/4 (100%)	0/5 (0%)	4/4 (100%)

G

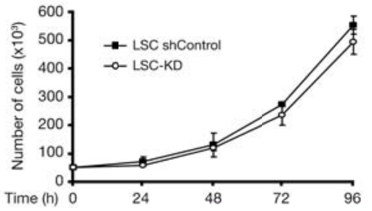

H

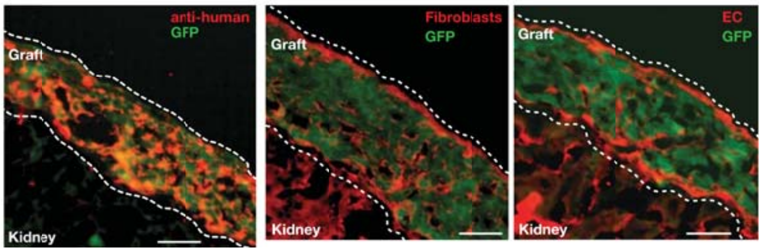

Supplementary Figure 3. SDF-1 is required for stromal cell recruitment. (A) Graph depicting the image tracing of LSC-shControl (left) or LSC lacking SDF-1 (LSC-KD) (right) cells coupled with fibroblasts in lung explants. Arrows indicate the starting points. (B) Graph showing the relative position of three different fibroblast/LSC-KD pairs (left) and the

depiction of their migration during 6 days (right) in lung explants. Arrows indicate the starting points. Note that in absence of SDF-1 expression, both cellular types migrate to opposite directions. (C) Comparison of SDF-1 mRNA levels in LSCs, LSC-KD and LSC-KD+SDF-1, the latter showing the rescue of SDF-1 levels by overexpression of mRNA resistant to shSDF-1 knockdown. The data are shown as the mean ± S.E.M. of 3 triplicate experiments. $P<0.01$ values were analysed by One-way ANOVA. (D) Graphs showing selected pairs of fibroblast/LSC-KD+SDF-1 (bottom) and the depiction of their migration during 7 days (top) in lung explants. Note that fibroblasts recruitment is rescued by overexpression of a SDF-1 mRNA resistant to shSDF-1 knockdown. Arrows indicate the starting points. (E) Kidney engraftments of LSCs (expressing a shControl), LSC-KD, or LSC-KD+SDF-1 (recombinant SDF-1, embedded in the matrigel, was used to inject the cells), at 2 weeks after injection under the renal capsule. (F) Table shows the number of mice injected and the positive engraftments in the kidney. (G) Knocking down SDF-1 in LSCs did not affect their proliferation. The data are shown as the mean ± S.E.M. of 4 triplicate experiments. (H) Labelling of injected human LSC-KD+SDF-1 cells (GFP-labelled) in kidney grafts using a specific anti-human mitochondrial antibody (left panel). Immunofluorescence in serial sections showing that LSCs induce the recruitment of fibroblasts (Vimentin^{+}, middle panel) and endothelial cells (CD73^{+}, right panel). Scale bars: 200μm.

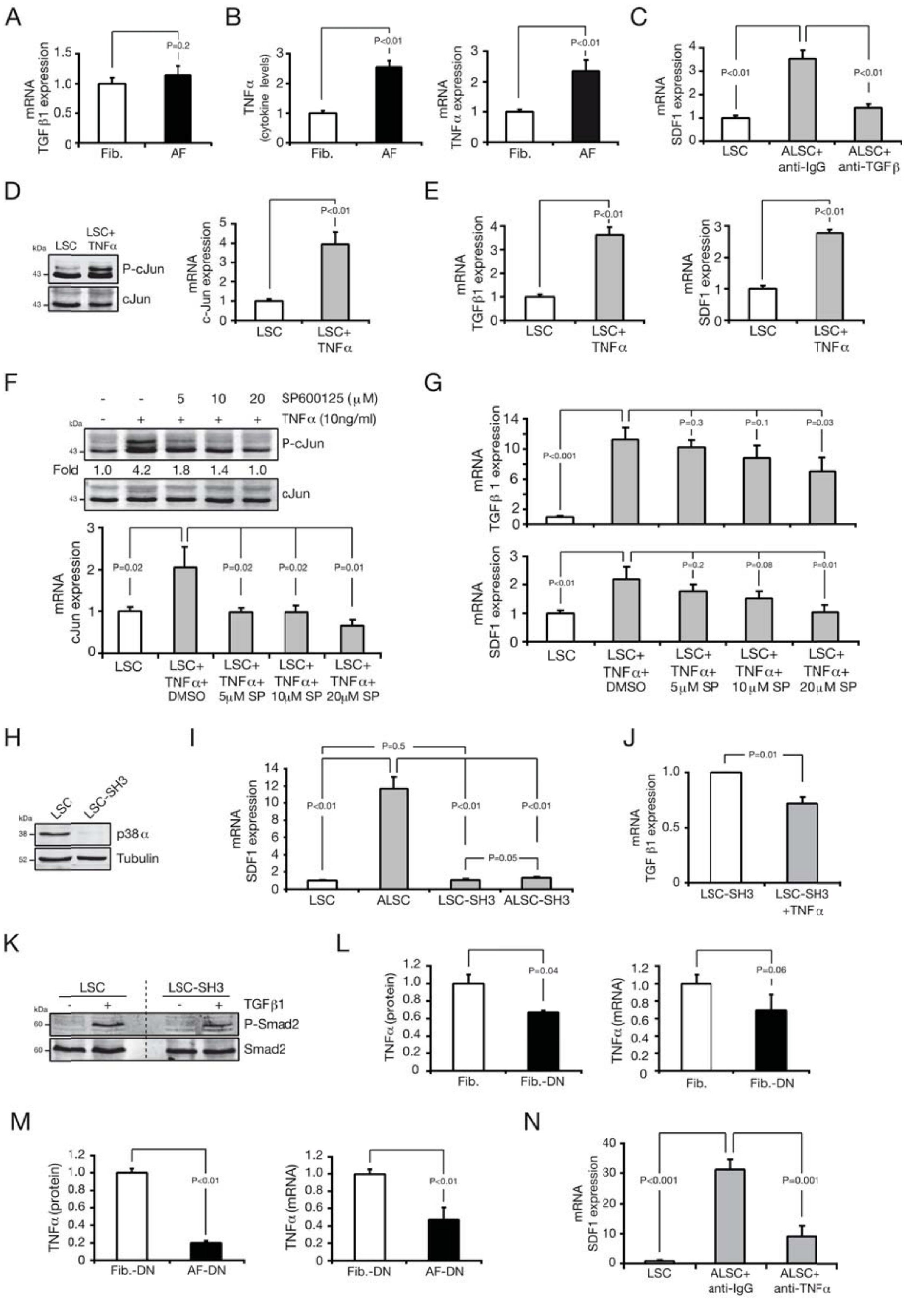
A
TGFβ1 expression
mRNA
P=0.2
Fib.
AF
B
TNFα (cytokine levels)
P<0.01
TNFα expression
mRNA
C
SDF1 expression
LSC
ALSC+ anti-IgG
ALSC+ anti-TGFβ
D
LSC
LSC+ TNFα
P-cJun
cJun
c-Jun expression
E
F
SP600125 (μM)
TNFα (10ng/ml)
Fold 1.0 4.2 1.8 1.4 1.0
cJun expression
P=0.02
P=0.01
LSC+ TNFα+ DMSO
LSC+ TNFα+ 5μM SP
LSC+ TNFα+ 10μM SP
LSC+ TNFα+ 20μM SP
G
P<0.001
P=0.3
P=0.1
P=0.03
P=0.2
P=0.08
H
LSC-SH3
p38α
Tubulin
I
P=0.5
P=0.05
ALSC
ALSC-SH3
J
LSC-SH3 +TNFα
K
TGFβ1
P-Smad2
Smad2
L
TNFα (protein)
TNFα (mRNA)
P=0.04
P=0.06
Fib.-DN
M
AF-DN
N
ALSC+ anti-TNFα
P=0.001

Supplementary Figure 4. Regulation of SDF-1 and TNFα expression. (A) Comparative TGFβ mRNA expression in fibroblasts and AFs. (B) TNFα protein levels and mRNA expression in fibroblasts and AFs. (C) SDF-1 expression in ALSCs can be inhibited with an anti-TGFβ blocking antibody. (D) Activation of cJun (P-cJun) and AP-1 targets (cJun itself) by TNFα in LSCs. (E) Recombinant TNFα induces TGFβ and SDF-1 mRNA expression in LSCs. (F) TNFα-induced AP-1 activity through JNK pathway was confirmed by adding a JNK small molecule inhibitor (SP600125) in a dose-dependent manner. (G) Inhibition of JNK pathway prevented SDF-1 expression induced by TNFα but only partially TGFβ expression. (H) Western-blot showing the knockdown of p38α protein in LSCs expressing a short hairpin against p38α (LSC-SH3). (I) SDF-1 expression in activated LSCs (ALSC) is abrogated in ALSC-SH3 cells lacking p38α activity. (J) TNFα-induced TGFβ expression is dependent on p38α signalling. (K) Activation of the Smad2 pathway by TGFβ is only marginally reduced in absence of p38α. (L) Comparative TNFα protein levels and mRNA expression between WT and $p38^{DN}$ fibroblasts. (M) TNFα protein and mRNA levels in fibroblasts or activated fibroblasts lacking p38α signalling. (N) SDF-1 expression in ALSCs induced by fibroblasts is initiated by TNFα and can be inhibited with an anti-TNFα blocking antibody. All results (Fig. A-G, I-J, L-N) are the mean ± S.E.M. of 4-5 triplicate experiments. P<0.01 values were defined as statistically significant, as analysed by One-way ANOVA.

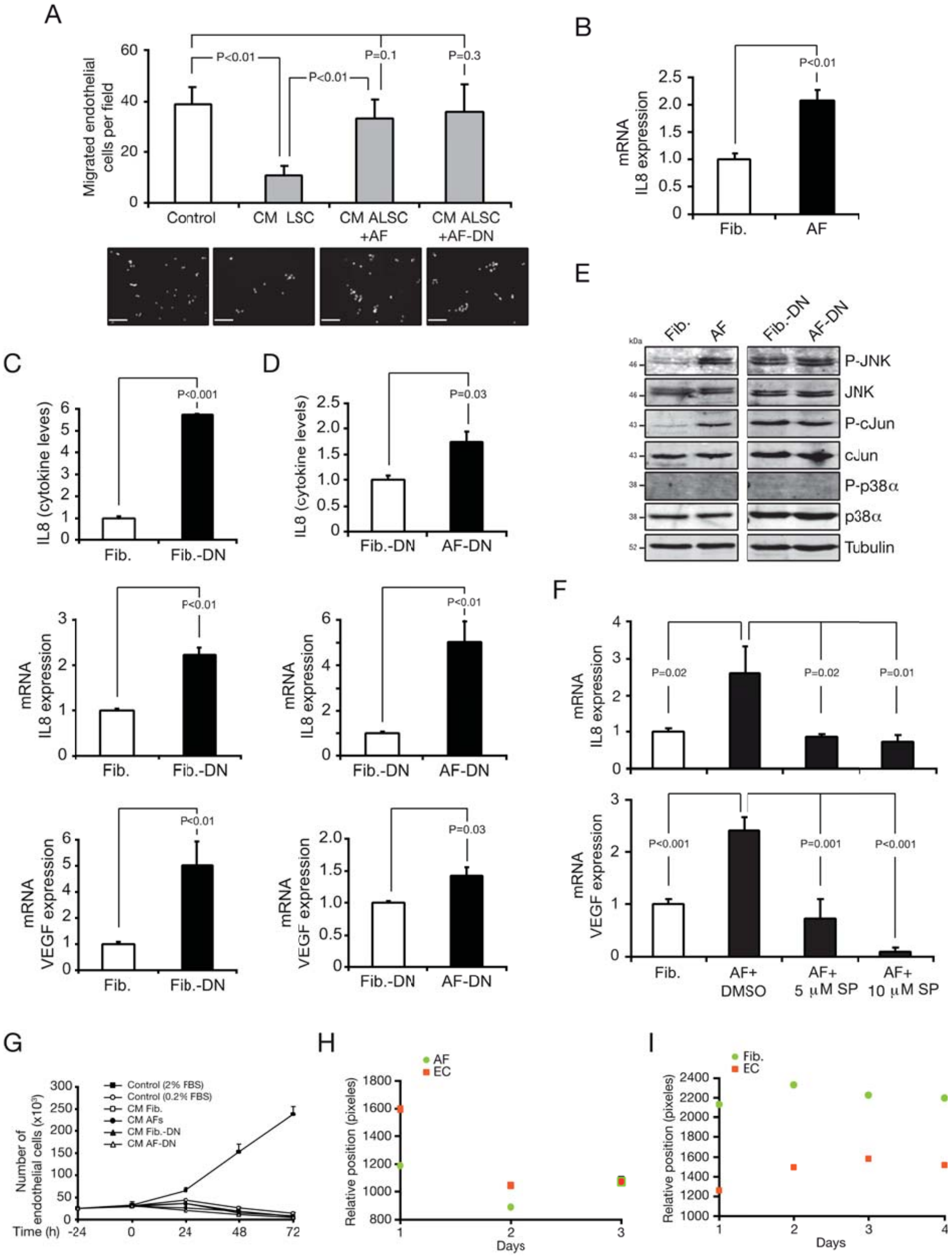

Supplementary Figure 5. Endothelial cell migration is mediated by angiogenic signals, induced by the JNK/AP-1 pathway in AFs. (A) Conditioned medium (CM) from LSCs

cultured alone (LSC), co-cultured with either WT (ALSC+AF) or p38^{DN} fibroblasts (ALSC+AF-DN), did not induce migration of endothelial cells in transwell chambers compared to the control (0.2% FBS). All results are the mean of 4 triplicate experiments ± S.E.M. Scale bars: 200μm. (B) IL8 mRNA expression is enhanced in activated fibroblasts (AF). (C) IL8 protein and mRNA levels and VEGF expression are increased in fibroblasts lacking p38α signalling. (D) IL8 and VEGF expression is induced in activated fibroblasts independently of p38α signalling (AF-DN). (E) Western-blot showing activation of JNK/AP-1 pathway at 12 hours in WT activated fibroblasts (AF) (left panel). Deficient p38α signalling activates the JNK/AP-1 pathway in fibroblasts (right panel). (F) JNK-dependent IL8 and VEGF expression was confirmed using a small chemical inhibitor of JNK (SP600125). (G) Endothelial cell proliferation is not promoted by CM from fibroblasts or AF with or without p38α signalling. (H and I) Fibroblasts and endothelial cells were injected into lung explants and then cell tracking was performed. Graphs of relative positioning of fibroblasts and endothelial cells show that (H) activated fibroblasts (AF) but not non-activated fibroblasts (I) can recruit endothelial cells in lung explants. The data (Fig. A-D, F-G) are shown as the mean ± S.E.M. of 4-5 triplicate experiments. $P<0.01$ values were defined as statistically significant, as analysed by One-way ANOVA.

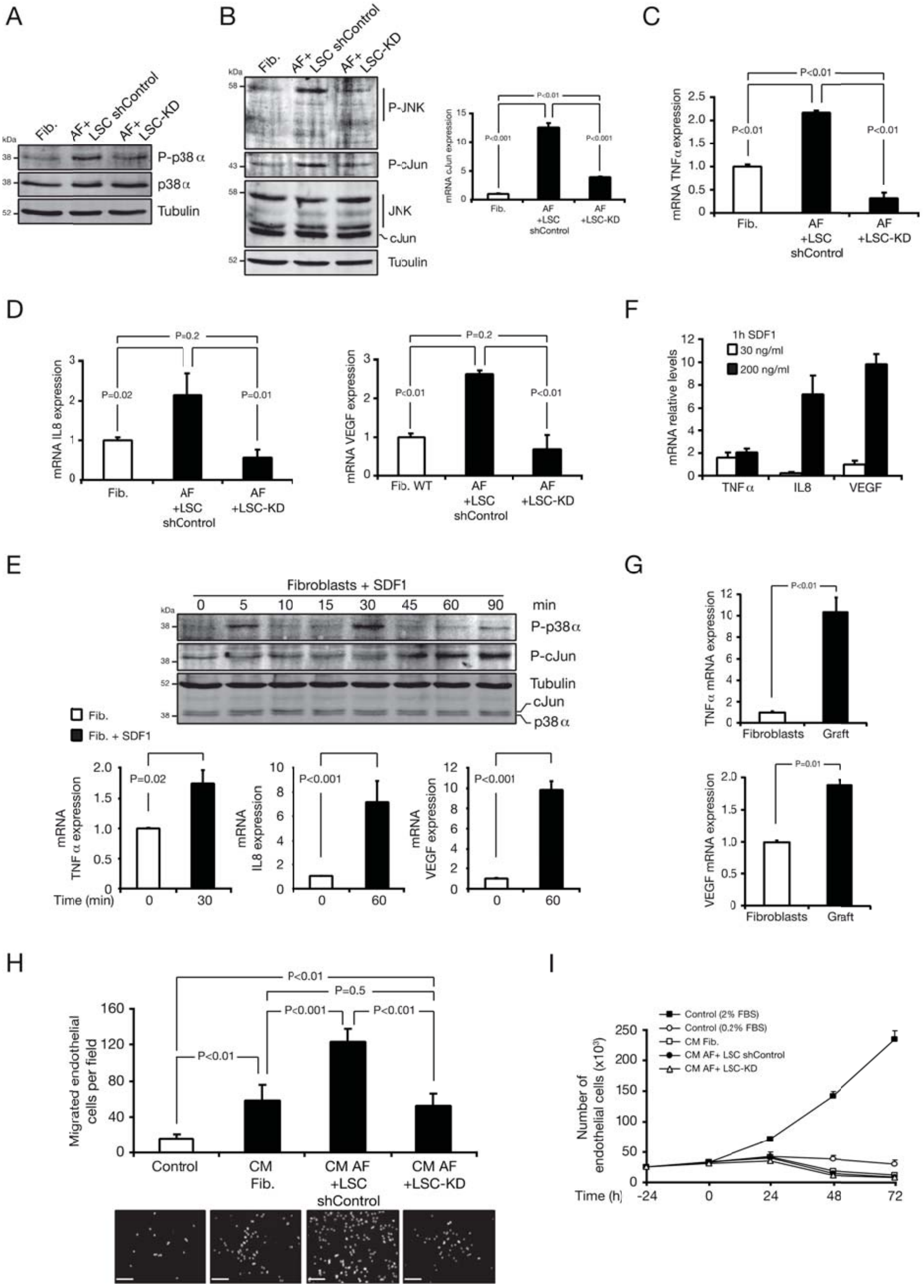
A
B
C
Fib.
AF+ LSC shControl
AF+ LSC-KD
kDa
P-p38α
p38α
Tubulin
P-JNK
P-cJun
JNK
cJun
Tubulin
mRNA cJun expression
P<0.01
P<0.001
P<0.001
Fib.
AF +LSC shControl
AF +LSC-KD
mRNA TNFα expression
P<0.01
P<0.01
P<0.01
D
mRNA IL8 expression
P=0.2
P=0.02
P=0.01
mRNA VEGF expression
P=0.2
P<0.01
P<0.01
Fib. WT
F
1h SDF1
30 ng/ml
200 ng/ml
mRNA relative levels
TNFα
IL8
VEGF
E
Fibroblasts + SDF1
0 5 10 15 30 45 60 90
min
P-p38α
P-cJun
Tubulin
cJun
p38α
Fib.
Fib. + SDF1
mRNA TNF α expression
P=0.02
mRNA IL8 expression
P<0.001
mRNA VEGF expression
P<0.001
Time (min)
G
TNFα mRNA expression
P<0.01
Fibroblasts
Graft
VEGF mRNA expression
P=0.01
H
P<0.01
P=0.5
P<0.001
P<0.001
P<0.01
Migrated endothelial cells per field
Control
CM Fib.
CM AF +LSC shControl
CM AF +LSC-KD
I
Control (2% FBS)
Control (0.2% FBS)
CM Fib.
CM AF+ LSC shControl
CM AF+ LSC-KD
Number of endothelial cells (x10³)
Time (h)

Supplementary Figure 6. SDF-1 controls TNFα and angiogenic factors expression in fibroblasts. (A) LSCs lacking SDF-1 (LSC-KD) failed to induce p38α and (B) JNK/AP-1 activation in fibroblasts (AF) after 6 and 12 hours of co-culture, respectively. (C) Knocking-down SDF-1 in LSCs abrogated paracrine fibroblast induction and expression of TNFα and (D) angiogenic factors (IL8, VEGF). (E) Upper panel: Western-blot showing activation of p38α (early activation, 30 min) and JNK/AP-1 (late activation, 60-90 min) pathways in fibroblasts induced by recombinant SDF-1 protein. Lower panel: Relative mRNA expression of TNFα, IL8 and VEGF in fibroblasts induced by recombinant SDF-1 protein. (F) Relative mRNA expression of TNFα, IL8 and VEGF in fibroblasts induced by different concentrations of recombinant SDF-1 protein after 1 hour. (G) Real-time qPCR analysis showing that TNFα and VEGF mRNA expression were up-regulated in kidney engraftments in comparison to mouse fibroblasts. (H) Only conditioned medium (CM) from AF co-cultured with LSCs (expressing control shRNA), but not CM from AF co-cultured with LSCs lacking SDF-1 (LSC-KD), could induce endothelial cell migration in transwell chambers. Scale bars: 200μm. (I) CM from AF co-cultured with either LSCs or LSC-KD did not induce changes in endothelial cell proliferation. 2% FBS is a positive control for endothelial proliferation. The data (Fig. B-I) are shown as the mean ± S.E.M. of 4-5 triplicate experiments. $P<0.01$ values were defined as statistically significant, as analysed by One-way ANOVA.

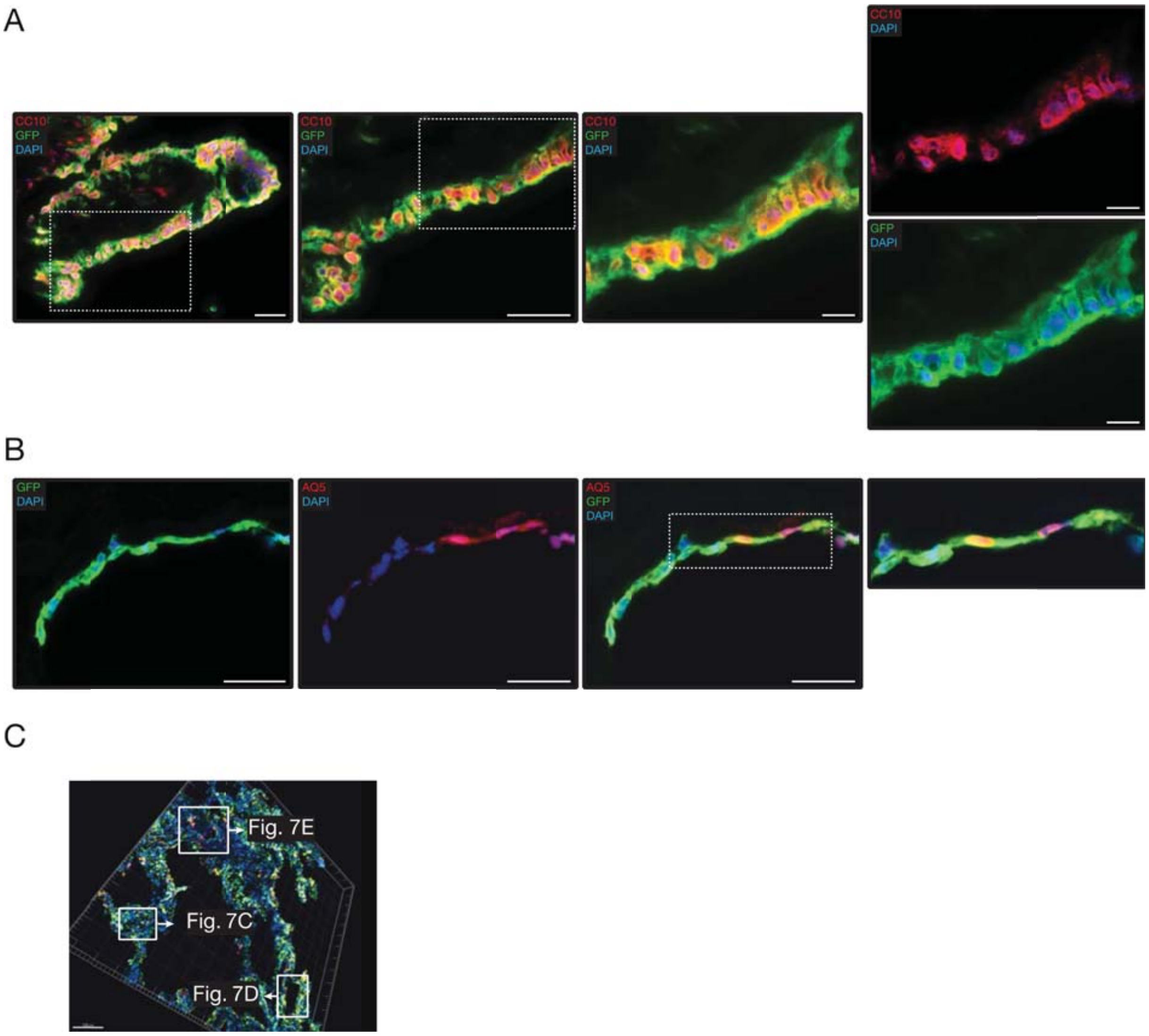

Supplementary Figure 7. LSCs differentiate in bronchiolar or alveolar cells. (A) Differentiation of LSCs in either bronchiolar Clara cells (CC10^{+}) or (B) in elongated alveolar type-I cells (AQ5^{+}) in lung explants. Scale bars: 50µm. (C) 3D-section of serial confocal images showing the LSC repopulation (green) of an *ex vivo* lung explant and the location of fibroblast foci (red). Squares indicate the region showed in figure 7C, 7D or 7E. Scale bars: 200µm.

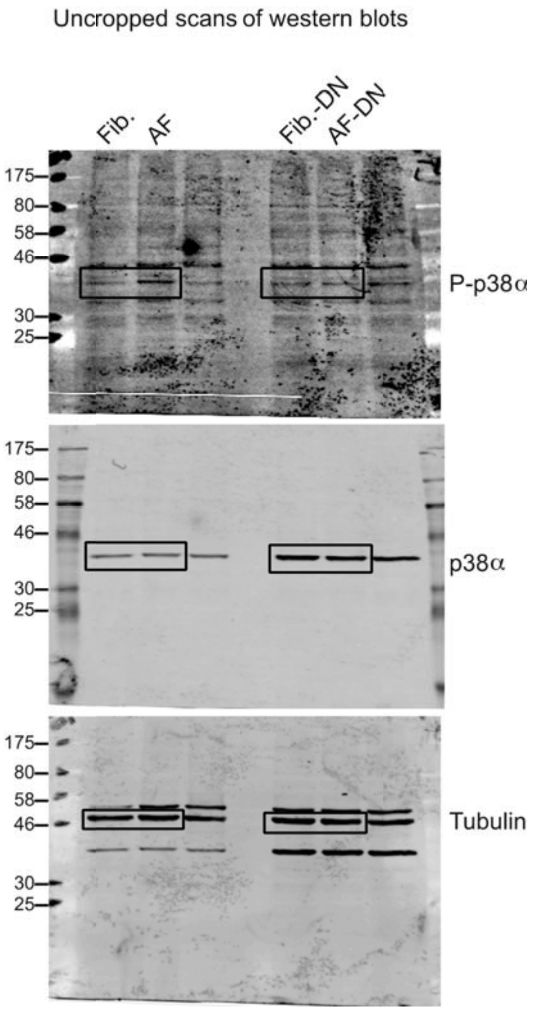

Supplementary Figure 8. Uncropped scans of p38a expression. P-p38a (phospho-p38a), p38a and Tubulin protein expression in Fibroblasts (Fib), Activated-LSCs (ALSC), and p38a-dominant negative (DN) counterparts.

Supplementary Table 1. LSCs protein array.

	LSC	ALSC	SD
BLC/CXCL13/BCA-1	1	1.2	0.17
C5A	1	1.4	0.04
G-CSF	1	0.9	0.24
GM-CSF	1	1.2	0.05
I-309/ CCL1/TCA-3	1	0.9	0.04
Eotaxin/CCL11	1	1.1	0.21
sICAM-1	1	1.2	0.05
IFN-γ	1	1.3	0.14
IL-1α	1	1.0	0.09
IL-1β	1	1.0	0.06
IL-1ra	1	0.9	0.09
IL-2	1	1.0	0.18
IL-3	1	1.1	0.04
IL-4	1	1.5	0.03
IL-5	1	1.0	0.12
IL-6	1	1.1	0.04
IL-7	1	1.2	0.27
IL-10	1	0.8	0.04
IL-13	1	1.1	0.03
IL-12 p70	1	1.4	0.09
IL-16	1	0.8	0.03
IL-17	1	1.3	0.30
IL-23	1	0.9	0.06
IL-27	1	0.6	0.08
CXCL10/CRG2	1	1.0	0.10
I-TAC/CXCL11	1	1.2	0.21
IL8/KC	1	2.0	0.09
M-CSF	1	2.1	0.29
CCL2/MCP1	1	3.1	0.09
MCP-5/CCL12	1	0.8	0.04
MIG/CXCL9	1	1.0	0.04
MIP-1α/ CCL3	1	1.2	0.02
MIP-1β/ CCL4	1	0.9	0.01
MIP-2	1	1.5	0.15
RANTES/CCL5	1	1.0	0.23
SDF-1/ CXCL12	1	2.5	0.31
TARC/ CCL17	1	0.9	0.21
TIMP-1	1	4.3	0.23
TNF-α	1	0.6	0.05
TREM-1	1	1.3	0.12

Comparative levels of cytokines released to the media by LSCs or ALSCs (co-cultured with fibroblasts).

Supplementary Table 2. Fibroblasts protein array.

	Fibroblasts	AF	SD
BLC/CXCL13/BCA-1	1	1.5	0.37
C5A	1	1.0	0.09
G-CSF	1	1.1	0.17
GM-CSF	1	1.2	0.10
I-309/ CCL1/TCA-3	1	0.9	0.12
Eotaxin/CCL11	1	1.9	0.49
sICAM-1	1	1.2	0.07
IFN-γ	1	0.7	0.03
IL-1α	1	1.4	0.04
IL-1β	1	1.6	0.04
IL-1ra	1	0.3	0.15
IL-2	1	1.6	0.08
IL-3	1	1.7	0.26
IL-4	1	1.0	0.06
IL-5	1	0.7	0.11
IL-6	1	1.2	0.08
IL-7	1	0.9	0.06
IL-10	1	0.9	0.11
IL-13	1	0.5	0.20
IL-12 p70	1	0.9	0.37
IL-16	1	0.8	0.14
IL-17	1	1.0	0.29
IL-23	1	1.7	0.21
IL-27	1	1.4	0.39
CXCL10/CRG2	1	1.1	1.20
I-TAC/CXCL11	1	1.1	0.04
IL8/KC	1	2.6	0.18
M-CSF	1	1.2	0.11
CCL2/MCP1	1	2.3	0.23
MCP-5/CCL12	1	1.0	0.14
MIG/CXCL9	1	0.9	0.02
MIP-1α/ CCL3	1	0.7	0.02
MIP-1β/ CCL4	1	1.1	0.14
MIP-2	1	1.5	0.02
RANTES/CCL5	1	1.0	0.16
SDF-1/ CXCL12	1	0.2	0.02
TARC/ CCL17	1	1.0	0.00
TIMP-1	1	1.7	0.10
TNF-α	1	2.3	0.17
TREM-1	1	0.6	0.27

Normalized levels of cytokines released to the media by AF (fibroblasts co-cultured with LSCs) compared to fibroblasts cultured alone.

Supplementary Table 3. Kidney graft assays at 2 weeks.

	LSC shControl	LSC-KD	LSC-KD +SDF-1
No. Engrafments (%)	4/4 (100%)	0/5 (0%)	4/4 (100%)

Kidney capsule injections and positive grafts obtained by LSCs, LSC-KD or LSC-KD+SDF-1 (rescued by overexpression of a SDF-1 mRNA resistant to shSDF-1 knockdown).

Supplementary Table 4. Kidney capsule assays at 4 weeks.

	LSC shControl	LSC-SH3	LSC-SH3 +SDF-1
No. Engrafments (%)	4/5 (80%)	0/7 (0%)	0/6 (0%)

Number of injected mice and positive engraftments in kidney capsule assays for LSCs (expressing shRNA control), LSC-SH3 (deficient in p38α) or LSC-SH3+SDF-1 (overexpression of a SDF-1 mRNA).

Supplementary Table 5. Real-time PCR primer sequences (5' to 3')

Target	Forward primer	Reverse primer
GAPDH	GAAGGTGAAGGTCGGAGT	GAAGATGGTGATGGGATTTC
SDF1α	TGAGAGCTCGCTTTGAGTGA	GTGGATCGCATTTATGCATG
SDF1β	CTAGTCAAGTGCGTCCACGA	GGACACACCACAGCACAAAC
TGF-β1	GAGCCTGAGGCCGACTACTA	CGGAGCTCTGATGTGTTGAA
TNF-α	TCATGCACCACCATCAAGGA	GACATTCGAGGCTCCAGTGAA
IL8	GCACCCAAACCGA AGT CATA	TGGGGACACCTTTTAGCATC
c-Jun	CCCCAAGATCCTGAAACAGA	CCGTTGCTGGACTGGATTAT
CCL2	TTGTCACCAAGCTCAAGAGAGA	GAGGTGGTTGTGGAAAAGGTAG
VEGF	TTACTGCTGTACCTCCACC	ACAGGACGGCTTGAAGATG
CXCR4	AGCTGTTGGCTGAAAAGCTGGTCTATG	GCGCTTCTGGTGGCCCTTGGAGTGTG
CXCR7	GGAAGCCCTGAGGTCACTTG	TTAGGCATGGTGGGACACTG
